# Diagnostic and Prognostic Accuracy of Cardiorespiratory Fitness for Detecting Cardiovascular Risk and Mitochondrial Disorders: An Overview of Systematic Reviews with Meta-Analyses

**DOI:** 10.1007/s40279-026-02503-7

**Published:** 2026-08-04

**Authors:** Brooklyn J. Fraser, Bethany Gower, Oliver Stanesby, Costan G. Magnussen, Cristina Cadenas-Sanchez, Jean-Philippe Chaput, Taru Manyanga, Ryan McGrath, Katherine Merucci, Jonathan Myers, Francisco B. Ortega, Ben Singh, Stephanie A. Prince, Justin J. Lang, Grant R. Tomkinson

**Affiliations:** 1 Menzies Institute for Medical Research, University of Tasmania, Private Bag 23, Hobart, TAS 7001 Australia; 2 Alliance for Research in Exercise, Nutrition and Activity (ARENA), School of Allied Health and Human Performance, Adelaide University, Adelaide, SA Australia; 3 Baker Heart and Diabetes Institute, Melbourne, VIC Australia; 4 Research Centre of Applied and Preventive Cardiovascular Medicine, University of Turku, Turku, Finland; 5 Centre for Population Health Research, University of Turku and Turku University Hospital, Turku, Finland; 6 Department of Physical Education and Sports, Faculty of Sport Sciences, Sport and Health University Research Institute (iMUDS), University of Granada, Granada, Spain; 7 Department of Cardiology, Stanford University, Stanford, CA USA; 8 Veterans Affairs Palo Alto Health Care System, Palo Alto, CA USA; 9 Centro de Investigación Biomédica en Red Fisiopatología de la Obesidad y Nutrición (CIBERobn), Instituto de Salud Carlos III, Granada, Spain; 10 Children’s Hospital of Eastern Ontario Research Institute, Ottawa, ON Canada; 11 Department of Pediatrics, Faculty of Medicine, University of Ottawa, Ottawa, ON Canada; 12 Division of Medical Sciences, University of Northern British Columbia, Prince George, BC Canada; 13 Healthy Aging North Dakota (HAND), North Dakota State University, Fargo, ND USA; 14 Department of Health, Nutrition, and Exercise Sciences, North Dakota State University, Fargo, ND USA; 15 Fargo VA Healthcare System, Fargo, ND USA; 16 Department of Geriatrics, University of North Dakota, Grand Forks, ND USA; 17 Health Library, Health Canada, Ottawa, ON Canada; 18 Faculty of Sport and Health Sciences, University of Jyväskylä, Jyväskylä, Finland; 19 Centre for Surveillance and Applied Research, Public Health Agency of Canada, Ottawa, ON Canada; 20 School of Epidemiology and Public Health, Faculty of Medicine, University of Ottawa, Ottawa, ON Canada

## Abstract

**Background:**

Cardiorespiratory fitness (CRF) is a powerful predictor of current and future health across all age groups. Recognising CRF’s ability to screen health-related outcomes is essential for supporting its use in clinical practice.

**Objective:**

The aim was to evaluate the health-related diagnostic and prognostic accuracy of CRF using an overview of systematic reviews.

**Methods:**

Five bibliographic databases (MEDLINE, Embase, Scopus, CINAHL and SPORTDiscus) were searched from January 2002 to April 2025 to identify systematic reviews with meta-analyses that reported on the health-related diagnostic and prognostic accuracy of CRF. The results were presented using forest plots, with the certainty of evidence assessed by a modified Grading of Recommendations, Assessment, Development, and Evaluations (GRADE) approach.

**Results:**

Of the 10,796 papers identified, six systematic reviews with meta-analyses (*n* = 31,171 observations) were included. Of these, five examined cardiovascular disease, events, or risk, and one examined mitochondrial disorders. CRF was measured objectively or estimated by exercise as maximum or peak oxygen consumption, ventilatory efficiency or fluctuation, or exercise performance or tolerance. All included reviews were graded as having low to critically low quality. CRF demonstrated high prognostic accuracy for predicting severe cardiovascular events in adults with heart failure, with moderate certainty (diagnostic odds ratio [dOR] 4.1–8.1; area under the curve of a summary receiver operating characteristic curve [SROC AUC] 0.73–0.77), and moderate-to-high diagnostic accuracy for detecting coronary artery disease in adults with type 2 diabetes or with suspected coronary artery disease with very low certainty (dOR 2.3–7.6; SROC AUC 0.66–0.79), mitochondrial disorders among adults with high certainty (dOR 18.1–26.6; SROC AUC 0.70–0.83), and cardiovascular disease risk among apparently healthy children and adolescents with low certainty (dOR 3.6–5.7; SROC AUC 0.64–0.71).

**Conclusions:**

CRF detects cardiovascular disease and mitochondrial disorders and predicts cardiovascular events among adults with chronic conditions and detects cardiovascular risk among apparently healthy children; however, the certainty of evidence ranged from very low to high certainty due to methodological limitations. Further research is needed to generate high-quality diagnostic evidence for CRF across diverse health outcomes and age groups in the general population.

**Trial Registration:**

PROSPERO CRD42022370149.

**Supplementary Information:**

The online version contains supplementary material available at https://doi.org/10.1007/s40279-026-02503-7.

## Key Points

**Table:** 

Six systematic reviews with meta-analyses examined the health-related diagnostic and prognostic accuracy of cardiorespiratory fitness for detecting cardiovascular disease, events, or risk (*n* = 5), and mitochondrial disorders (*n* = 1).
Cardiorespiratory fitness demonstrates moderate-to-high diagnostic accuracy for detecting cardiovascular risk among apparently healthy children and cardiovascular disease and mitochondrial disorders among adults with chronic conditions, and high prognostic accuracy for predicting cardiovascular events among adults with heart failure, with certainty of evidence ranging from very low to high due to methodological limitations in the summarised reviews.
Additional high-quality evidence for the diagnostic and prognostic accuracy of cardiorespiratory fitness in the detection of diverse health outcomes in the general population is needed.

## Introduction

Cardiorespiratory fitness (CRF) is a health-related component of physical fitness that refers to the overall capacity of the circulatory and respiratory systems to supply and use oxygen for energy transfer to support muscle activity during large muscle, rhythmic, continuous physical activity [1]. CRF is widely expressed as maximal or peak oxygen consumption (or $\dot{V}$O_2peak_), which can be objectively measured (e.g. maximal exercise testing with gas analysis), estimated from exercise performance (e.g. graded treadmill or cycle ergometer protocols), or estimated without exercise (i.e. validated algorithms based on demographic and health indicators such as age, sex, resting heart rate) [2]. However, CRF is a multidimensional construct encompassing physiologically distinct components of cardiorespiratory and metabolic function. Because $\dot{V}$ O_2max_ alone does not describe all aspects of CRF [3], other measures such as exercise ventilatory efficiency (e.g. ventilation relative to carbon dioxide production) are also used [4].

The health benefits of higher CRF extend throughout the lifespan. In 2020, the American Heart Association endorsed CRF as an important marker of cardiovascular health among children and adolescents [5]. Low CRF in adults is an independent and strong risk factor for cardiovascular morbidity and early all-cause and cardiovascular mortality among both healthy and diseased populations [6]. Low CRF is also a stronger predictor of cardiovascular outcomes than established modifiable risk factors such as smoking, hypertension, or hyperlipidaemia [3]. Given the mounting evidence linking higher CRF among children [7] and adults [6] with improved cardiovascular health, the American Heart Association has advocated that CRF be used as a clinical vital sign for cardiovascular disease risk among adults  [3, 8].

Despite the well-established cardiovascular health benefits associated with high levels of CRF, it remains unclear whether CRF can identify individuals with cardiovascular disease (i.e. its diagnostic accuracy) or predict those at high risk for incident events or adverse outcomes (i.e. its prognostic accuracy, which supports risk stratification and longer-term management). A previous systematic review examined the health-related diagnostic accuracy of CRF in youth [9], with results indicating a high degree of variation in the health-related discriminatory utility of different CRF tests to support universal criterion-referenced cut points [9]. However, an overview of systematic reviews (also called an umbrella review), which synthesises data from published systematic reviews and meta-analyses to comprehensively assess topical evidence, provides one of the highest levels of research evidence [10]. Overviews meaningfully contribute to evidence-based practice and evidence-informed decision making by creating a broad topical perspective and identifying knowledge gaps that can inform future research [11]. Synthesising the breadth and quality of meta-analysed data on the diagnostic accuracy of CRF for various health outcomes will help inform future directions supporting CRF use in clinical practice. Treating CRF as an umbrella construct, consistent with its use in clinical and epidemiological research, enables synthesis of its broader health-related diagnostic accuracy without implying mechanistic equivalence across measures. Therefore, to better understand the diagnostic and prognostic accuracy of CRF across health outcomes, stratified by specific test modality and outcome, we conducted an overview of systematic reviews with meta-analyses.

## Methods

### Registration and Protocol

The protocol for this overview was registered with the International Prospective Register of Systematic Reviews (PROSPERO #CRD42022370149) and adhered to the Preferred Reporting Items for Overviews of Reviews (PRIOR) statement [12] and the Meta-analyses of Observational Studies in Epidemiology (MOOSE) reporting standards [13] (see Electronic Supplementary Material [ESM] eAppendix 1 for the PRIOR checklist). This overview is a sub-study of a larger overview of reviews project examining the associations between CRF and health outcomes across the lifespan. Two published sibling overviews evaluated the predictive associations between CRF and health outcomes separately among children and adolescents [7] and adults [6].

### Eligibility Criteria

In this overview, we included systematic reviews with meta-analyses that assessed the health-related diagnostic or prognostic accuracy of CRF. Studies were eligible if they met the following criteria:

- *Population:* Humans of any age from the general population or clinical populations with diagnosed chronic conditions.
- *Exposure:* CRF measured objectively (i.e. maximal exercise testing with gas analysis) or estimated by exercise (i.e. maximal or submaximal exercise testing without gas analysis) or non-exercise algorithms.
- *Outcome:* Provided diagnostic or prognostic accuracy to detect any health-related outcomes, including mortality, chronic health conditions and diseases, or mental health and wellbeing indicators.
- *Study design*: Systematic reviews with meta-analyses that searched at least two bibliographic databases, provided a sample search strategy, and meta-analysed primary diagnostic or prognostic accuracy studies.
- *Publication status and language restriction:* Full-text original systematic reviews with meta-analyses published in peer-reviewed journals. Based on the authors’ language capacity, only reviews published in English, French, or Spanish were eligible. Conference abstracts, commentaries, editorials, dissertations, or grey literature were ineligible.
- *Timeframe:* Systematic reviews with meta-analyses published after 1 January 2002 were included to capture the 20-year period from the initial search. No date restrictions were applied to the primary studies included in the systematic reviews.

### Information Sources and Search Strategy

A search of five bibliographic databases (MEDLINE^®^ [via Ovid], Embase [via Ovid], Scopus, CINAHL [via EBSCOhost], and SPORTDiscus [via EBSCOhost]) was originally conducted for eligible systematic reviews with meta-analyses published between 1 January 2002 and 18 November 2022. One search update was carried out from 1 November 2022 to 8 March 2024, and a second update was carried out from 1 February 2024 to 11 April 2025 to search for articles published since the preceding search. The search strategy was created by a library scientist (KM) in collaboration with the authors. An independent research librarian peer-reviewed the final search using the Peer Review of Electronic Search Strategies (PRESS) guidelines [14]. The search strategies for databases are available in ESM eAppendix 2. The reference lists of included reviews were also searched.

### Selection Process

Records were imported into RefWorks for deduplication before being imported into Covidence for further deduplication and screening. Reviewers were not blinded to the study metadata when screening. The following independent reviewers screened each title and abstract in duplicate against the eligibility criteria: JJL, SAP, CCS, JPC, BJF, TM, BS, BG, and GRT. Conflicts during title and abstract screening automatically progressed to full-text screening. Independent reviewers (JJL, SAP, CCS, JPC, BJF, TM, BS, BG, or GRT) screened full-text records in duplicate against the eligibility criteria. Subsequent conflicts were resolved through consensus-based discussion between two of the following reviewers: JJL, SAP, BJF, BG, or GRT. Conflicts were resolved by a third reviewer (BG or GRT).

### Data Collection Process and Data Items

Two investigators (BJF and OS) independently extracted relevant data, with discrepancies resolved by discussion. If required, additional information was requested by email by contacting the corresponding authors. The following descriptive data were extracted: author(s), year of publication, search strategy (e.g. search date range, databases searched), details of primary studies included in the meta-analysis (e.g. countries represented, number of primary studies), participant characteristics (e.g. age, sex, target population), CRF measure(s), health outcome(s), risk-of-bias results, and summary effect estimates from the meta-analyses (e.g. diagnostic odds ratio [dOR], area under the curve of a summary receiver operating characteristic curve [SROC AUC], sensitivity, specificity).

### Methodological Quality and Certainty of the Evidence Assessment

Two independent investigators (BJF and OS) separately assessed the methodological quality of each systematic review using A MeaSurement Tool to Assess systematic Reviews (AMSTAR 2) [15]. Conflicts were resolved by discussion. The risk-of-bias assessments (i.e. Quality Assessment of Diagnostic Accuracy Studies [QUADAS] and QUADAS-2 scores) for primary studies were extracted from the systematic reviews and used to inform the Grading of Recommendations, Assessment, Development, and Evaluations (GRADE) assessment. For each outcome, the certainty of the evidence was assessed by BG using a modified GRADE framework [16], and verified for accuracy by BJF or GRT. Disagreements were resolved through discussion until consensus was reached (see ESM eAppendix 4 for GRADE decision rules).

### Synthesis Methods and Data Analyses

Our goal was to identify systematic reviews with no to minimal overlapping primary studies for each outcome. If more than one systematic review examined the association between CRF and an outcome in the same target population, we calculated the corrected covered area (CCA) [17]. If the CCA was high (11–15%) or very high (> 15%), we retained the review with the highest quality rating (assessed using AMSTAR 2), or if review quality was equivalent, the most recent review.

The overall effect measures across the systematic reviews were summarised using a narrative synthesis. The primary effect measures of interest were dOR, SROC AUC, sensitivity, and specificity values. The dOR describes the odds of a positive test in those with disease relative to the odds in those without disease [18]. SROC AUC summarises pooled data across studies to describe the overall accuracy of the test to discriminate between those with and without disease. Sensitivity, or the true positive rate, describes the probability of a positive test in those with disease. Specificity, or the true negative rate, describes the probability of a negative test in those without disease. We reported the overall effect measures for each test and health outcome combination as described by the systematic review authors (e.g. for each health outcome, stratified effect statistics for multiple CRF measures were included). Summary effects are presented using forest plots. To interpret the magnitude of effect, dOR values of 2.0, 3.0, and 4.0 [19] and SROC AUC values of 0.56, 0.64, and 0.71 [20] were used as thresholds for low, moderate, and high diagnostic accuracy, respectively. Higher sensitivity supports the use of a test to rule out disease, whereas higher specificity indicates greater utility for ruling in disease.

### Deviation from Registered Protocol

We planned to only include meta-analyses that pooled data from primary cohort or case–control studies because of their ability to assess causality. However, we extended the search to include meta-analyses of primary cross-sectional and intervention studies to explore the diagnostic accuracy of CRF (for this review) and associations between CRF and health outcomes in children and adolescents (a sibling overview [6]).

## Results

### Study Selection

**Fig. 1:**
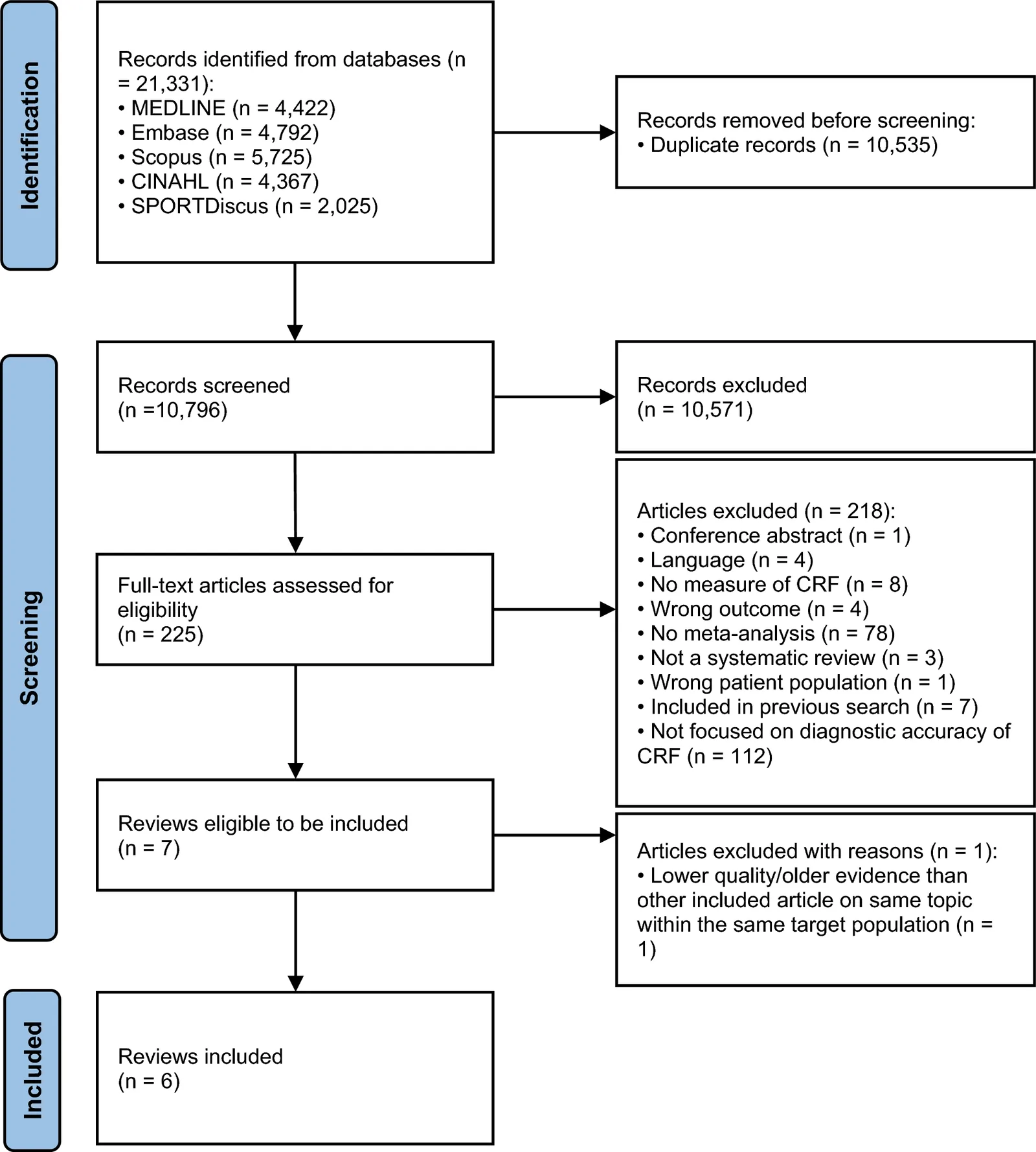
PRISMA flow diagram of the screening process. *CRF* cardiorespiratory fitness, *PRISMA* Preferred Reporting Items for Systematic reviews and Meta-Analyses

Figure 1 provides a flow diagram of the literature search and screening process, including reasons for full-text exclusion. Following the removal of duplicates, 10,796 records were screened at the title/abstract level, reducing to 225 full-texts screened (see ESM eAppendix for full texts with reasons for exclusion). Seven reviews [21–27] were retained after full-text screening. Complete summary statistics (dOR or SROC AUC, and sensitivity and specificity values) were provided in six reviews, with summary statistics not available for the seventh review [25]. As such, findings from the seventh review were included in our narrative overview but not in our forest plots. Two of the seven reviews [21, 27] examined the prognostic accuracy of CRF to predict severe cardiovascular events in patients with heart failure and had a high CCA value (CCA = 35%), with the results from the higher quality review retained [21]. Six systematic reviews were, therefore, included in this overview [21–26].

### Study Characteristics

Individual systematic reviews with meta-analysis characteristics are presented in Table 1. All meta-analyses were published in English between 2012 and 2024 and included 31,171 observations. The target population varied among meta-analyses, including those with systolic heart failure patients [21], apparently healthy children and adolescents [23], adults without known coronary artery disease [25], adult females with suspected and subclinical coronary artery disease [24], patients with type 2 diabetes without diagnosed coronary artery disease [22], and adults with suspected mitochondrial disorders. [26] Participant ages ranged from 8 to 19 years in the lone child and adolescent meta-analysis [23], with the mean age of participants ranging from 34 to 69 years across the five adult meta-analyses [21,22, 24–26]. The number of primary studies included in the meta-analyses ranged from seven to 30, with the number of observations in each meta-analysis ranging from 271 to 9280 participants.

**Table 1:** Overview of systematic review with meta-analysis characteristics

Study	Population description	Primary studies	Total sample	Sex	Age	CRF measure(s)	Health outcome	AMSTAR 2 category
Banerjee et al. [25]	Adults with symptoms suggestive of coronary artery disease, or who were asymptomatic but had risk factors for coronary artery disease	34	3352	NR	45–68 years (range of mean ages)	Exercise stress tests performed using: • Treadmill walking ECG (18/34) • Treadmill walking echocardiogram (9/34) • Cycle ergometry ECG (12/34) • Cycle ergometry echocardiogram (8/34) • Myocardial perfusion imaging (17/34)	Coronary artery disease detected via angiography, examination postmortem, or after long-term follow-up for the development of an acute coronary syndrome	Low
Cahalin et al. [21]	Adults with systolic heart failure (left ventricular ejection fraction < 50%) or a conformed congenital heart defect	30	$\dot{V}$O_2peak_: 7319	$\dot{V}$O_2peak_: 74% male	$\dot{V}$O_2peak_: 55 years (median)	$\dot{V}$O_2peak_ (19/30)	Severe cardiovascular events, defined as death, cardiac transplantation, implantation of a ventricular assist device, and/or myoplasty	Low
			$\dot{V}$_E_/$\dot{V}$CO_2_ slope: 5044	$\dot{V}$_E_/$\dot{V}$CO_2_ slope: 78.4% male	$\dot{V}$_E_/$\dot{V}$CO_2_ slope: 55 years (median)	$\dot{V}$_E_/$\dot{V}$CO_2_ (15/30)		
			EOV: 1617	EOV: 73% male	EOV: 58 years (median)	EOV (5/30)		
			OUES: 584	OUES: 85.1% male	OUES: 57.7 years (median)	OUES (2/30)		
Ruiz et al. [23]	Apparently healthy children and adolescents	7	9280	50.8% male	8–19 years (range)	$\dot{V}$O_2max_ estimated by: • 20-m shuttle run (3/7) • Submaximal treadmill walking (2/7) • Maximal cycle ergometry (2/7)	Cardiovascular disease risk defined as a clustering of risk factors or a continuous standardised cardiovascular risk score	Critically low
Sun et al. [24]	Women with suspected and subclinical coronary artery disease	19	2396	100% female	46.8–69 years (range of mean ages)	Treadmill exercise	Coronary artery disease detected via coronary angiography	Low
Dun et al. [22]	Adults with type 2 diabetes mellitus but without diagnosed coronary artery disease	8 (9 datasets)	515	51% male	48–60 years (range of mean ages)	Exercise stress tests performed using: • Treadmill exercise (3/9) • Cycle ergometry (6/9)	Coronary artery disease detected via coronary angiography	Low
El Guessabi et al. [26]	Adults with suspected mitochondrial disorders	14	Absolute load lactate stress test: 793	NR	34.2–58.0 years (range of mean ages)	Lactate stress tests using: • Absolute load lactate stress test protocol (6/14) • Relative load lactate stress test protocol (8/14)	Mitochondrial disorders detected via muscle specimens or genetic testing, with clinical subtypes including the following: CPEO, Kearns-Sayre syndrome, CPEO-plus, MERRF, LHON, PEO, unspecified subtypes of MCP, MELAS, NARP, POLG-related disorders, and Leigh syndrome	Low

*AMSTAR 2* A MeaSurement Tool to Assess systematic Reviews, *CPEO* chronic progressive external ophthalmoplegia, *CRF* cardiorespiratory fitness, *ECG* electrocardiogram, *EOV* exercise oscillatory ventilation, *LHON* Leber’s hereditary optic neuropathy, *MCP* mitochondriopathy, *MELAS* mitochondrial encephalomyopathy, lactic acidosis, and stroke-like episodes, *MERRF* myoclonic epilepsy with ragged-red fibres, *NARP* neuropathy, ataxia, and retinitis pigmentosa, *NR* not reported, *OUES* oxygen uptake efficiency slope, *PEO* progressive external ophthalmoplegia, *POLG *polymerase gamma,  $\dot{V}$_*E*_*/*$\dot{V}$*CO*_*2*_* slope* minute ventilation/carbon dioxide production slope, $\dot{V}$*O*_*2max*_ maximal oxygen consumption, $\dot{V}$*O*_*2peak*_ peak oxygen consumption

Collectively, these meta-analyses provided insight into the health-related diagnostic and prognostic accuracy [21–26] of CRF. There was a mix of CRF measures within and between the included meta-analyses, including objectively measured or exercise estimated $\dot{V}$O_2max_ or $\dot{V}$ O_2peak_ [21, 23], ventilatory efficiency (minute ventilation/carbon dioxide production slope [$\dot{V}$_E_/$\dot{V}$CO_2_ slope], oxygen consumption [$\dot{V}$O_2_] efficiency slope) [21], ventilatory fluctuation (exercise oscillatory ventilation) [21], aerobic exercise performance (via treadmill running/walking or cycle ergometry without concomitant gas analysis [22, 24], or treadmill running/walking or cycle ergometry with an electrocardiogram (ECG), echocardiogram, or myocardial perfusion imaging [25]) and exercise tolerance (absolute or relative load lactate stress tests [26]). Several cardiovascular health outcomes were considered in each meta-analysis, including severe cardiovascular events/death (defined as sudden cardiac death, cardiac transplantation, implantation of a ventricular assist device, and/or myoplasty [21]), clustered cardiovascular disease risk [23], and coronary artery disease [22, 24,25], with one review reporting on mitochondrial disorders. [26]

### Quality and Certainty of Evidence Assessment

The quality ratings of the included systematic reviews are presented in Table 1. Five of the six reviews were rated as having low quality [21,22, 24–26], and one was rated as having critically low quality [23]. The GRADE of the evidence is summarised in ESM eAppendix 4. Evidence for severe cardiovascular events was graded as moderate certainty due to some risk of bias and high heterogeneity. Evidence for cardiovascular disease risk was graded as low certainty due to risk of bias, high heterogeneity, and indirectness. Evidence for coronary artery disease was graded as very low certainty due to risk of bias, high heterogeneity, inconsistency, and indirectness. Evidence for mitochondrial disorders was graded as high certainty partially downgraded for indirectness but upgraded for a large magnitude of effect with no critical flaws identified.

### Synthesis of Results

**Fig. 2:**
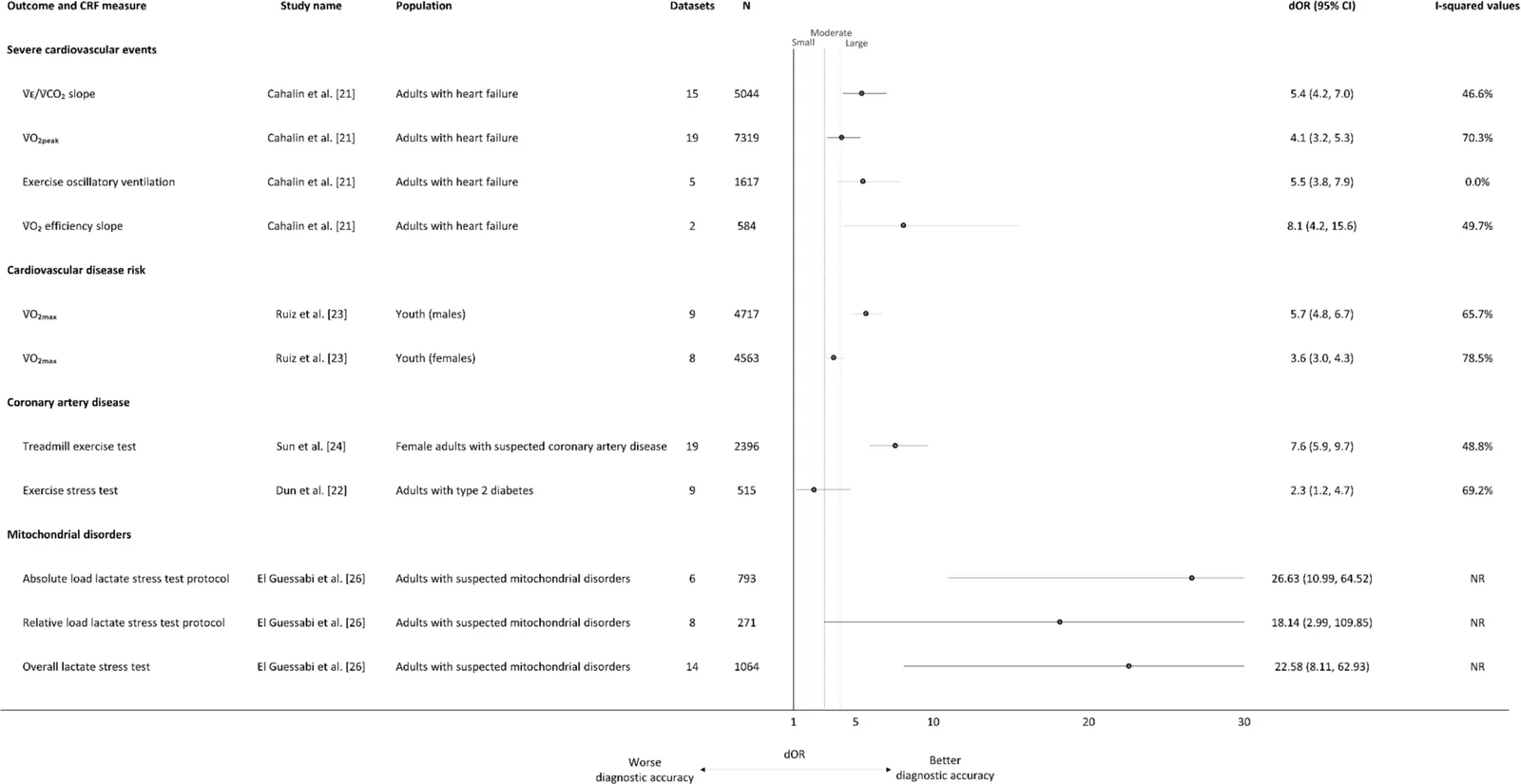
Diagnostic odds ratio (dOR) values stratified by outcome and cardiorespiratory fitness (CRF) measure. dOR describes the odds of a positive test in those with disease relative to the odds in those without disease. To interpret the magnitude of effect, dOR values of 2.0, 3.0, and 4.0^19^ were used as thresholds for low, moderate, and high diagnostic accuracy, respectively. Severe cardiovascular events were defined as sudden cardiac death, cardiac transplantation, implantation of a ventricular assist device, and/or myoplasty. The *x*-axis has been capped at 30 to enhance visualisation of results. *CI* confidence interval, *N* sample size, *NR* not reported, $\dot{V}$_*E*_*/*$\dot{V}$*CO*_*2*_* slope* minute ventilation/carbon dioxide production slope, $\dot{V}$*O*_*2*_ oxygen consumption, $\dot{V}$*O*_*2max*_ maximal oxygen consumption, $\dot{V}$*O*_*2peak*_ peak oxygen consumption

**Fig. 3:**
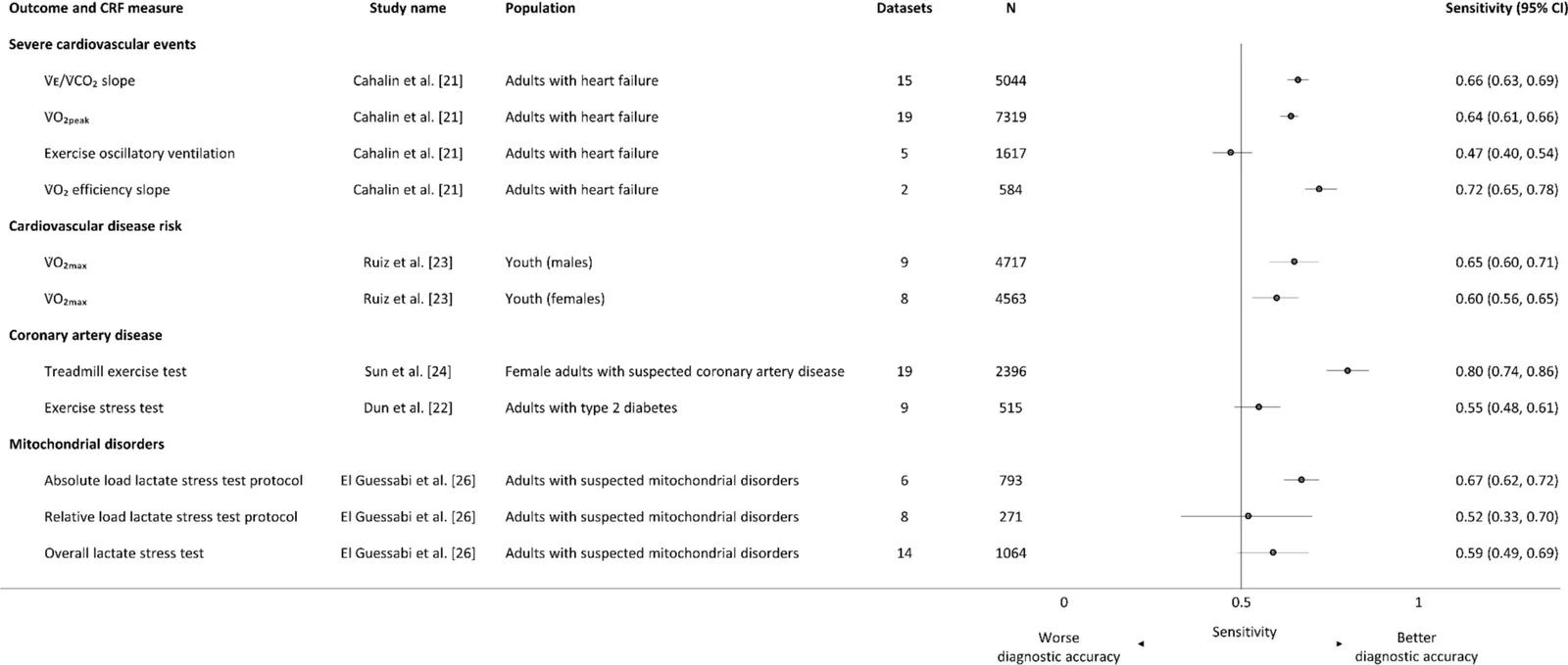
Sensitivity values stratified by outcome and cardiorespiratory fitness (CRF) measure. Sensitivity, or the true positive rate, describes the probability of a positive test in those with disease. Severe cardiovascular events were defined as sudden cardiac death, cardiac transplantation, implantation of a ventricular assist device, and/or myoplasty. *CI* confidence interval, *N* sample size, $\dot{V}$_*E*_*/*$\dot{V}$*CO*_*2*_* slope* minute ventilation/carbon dioxide production slope, $\dot{V}$*O*_*2*_ oxygen consumption, $\dot{V}$*O*_*2max*_ maximal oxygen consumption, $\dot{V}$*O*_*2peak*_ peak oxygen consumption

**Fig. 4:**
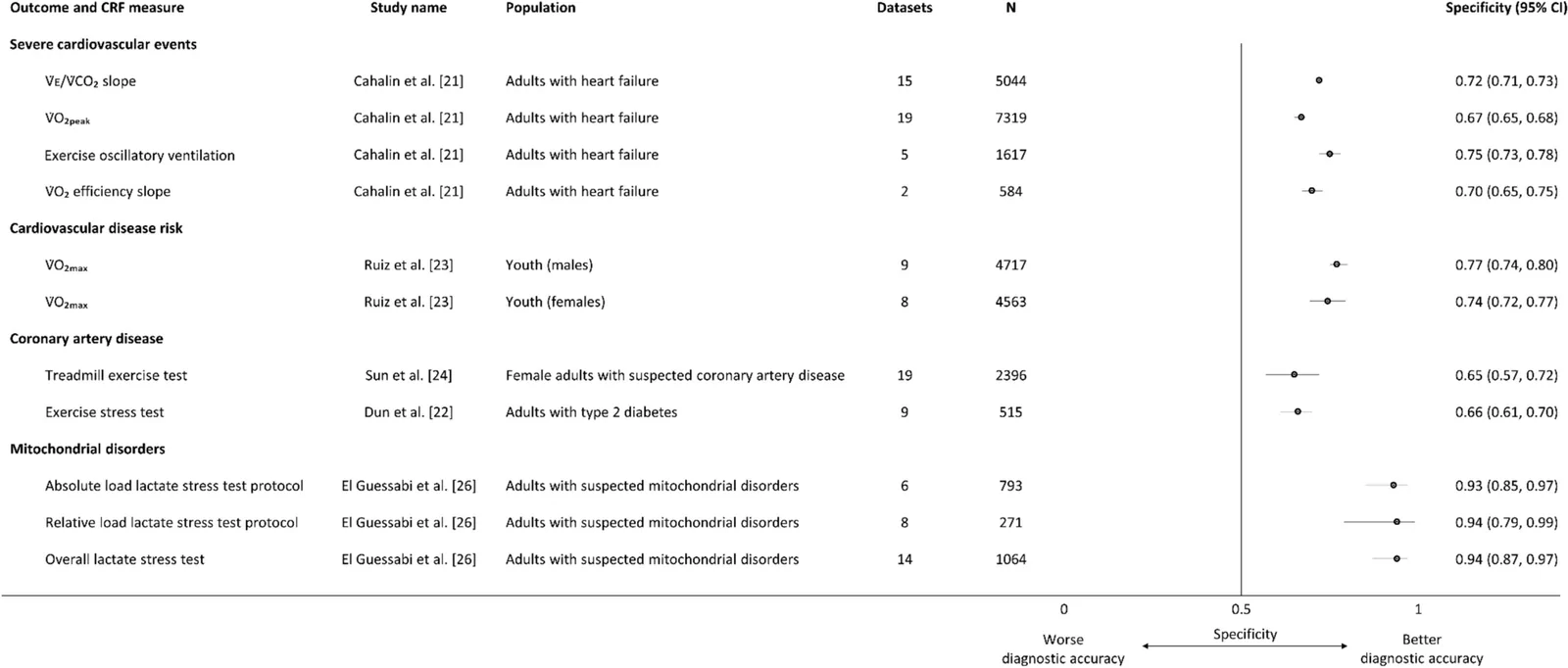
Specificity values stratified by outcome and cardiorespiratory fitness (CRF) measure. Specificity, or the true negative rate, describes the probability of a negative test in those without disease. Severe cardiovascular events were defined as sudden cardiac death, cardiac transplantation, implantation of a ventricular assist device, and/or myoplasty. *CI* confidence interval, *N* sample size, $\dot{V}$_*E*_*/*$\dot{V}$*CO*_*2*_* slope* minute ventilation/carbon dioxide production slope, $\dot{V}$*O*_*2*_ oxygen consumption, $\dot{V}$*O*_*2max*_ maximal oxygen consumption, $\dot{V}$*O*_*2peak*_ peak oxygen consumption

**Fig. 5:**
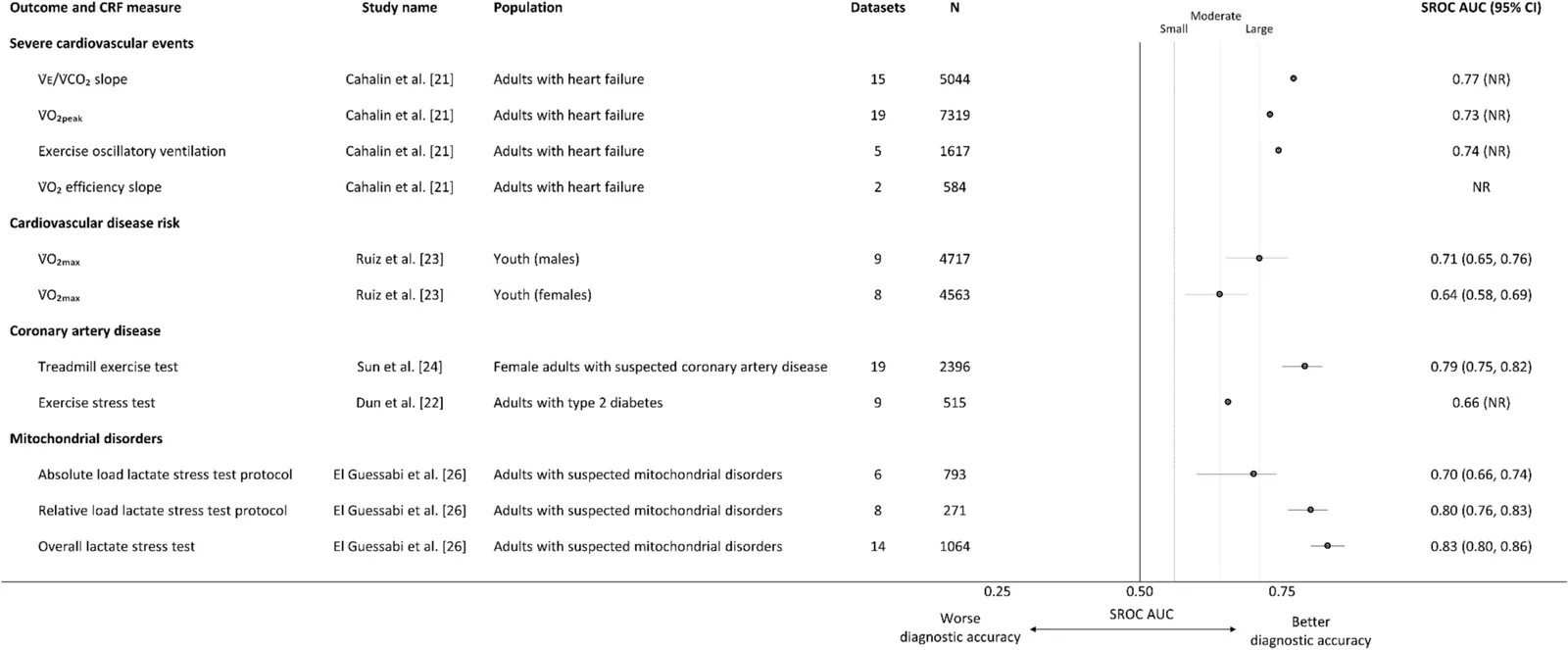
Area under the curve of a summary receiver operating characteristic curve (SROC AUC) values stratified by outcome and cardiorespiratory fitness (CRF) measure. SROC AUC summarises pooled data across studies to describe the overall accuracy of the test to discriminate between those with and without disease. To interpret the magnitude of effect, SROC AUC values of 0.56, 0.64, and 0.71^20^ were used as thresholds for low, moderate, and high diagnostic accuracy, respectively. Severe cardiovascular events were defined as sudden cardiac death, cardiac transplantation, implantation of a ventricular assist device, and/or myoplasty. *CI* confidence interval, *N* sample size, *NR* not reported, $\dot{V}$_*E*_*/*$\dot{V}$*CO*_*2*_* slope* minute ventilation/carbon dioxide production slope, $\dot{V}$*O*_*2*_ oxygen consumption, $\dot{V}$*O*_*2max*_ maximal oxygen consumption, $\dot{V}$*O*_*2peak*_ peak oxygen consumption

The dOR, sensitivity, specificity, and SROC AUC results included in each meta-analysis stratified by health outcome are presented in Figs. 2, 3, 4, and 5. An overview of findings from the included meta-analyses is provided within this sub-section.

#### Prognostic Accuracy Among Adults

##### Severe Cardiovascular Events

There was moderate certainty of evidence for the ability of CRF, defined as $\dot{V}$_E_/$\dot{V}$CO_2_ slope, $\dot{V}$O_2peak_, exercise oscillatory ventilation, or $\dot{V}$O_2_ efficiency slope, to predict severe cardiovascular events in adults with heart failure, as per findings from one meta-analysis [21]. The dOR, sensitivity, and specificity values were similar for most CRF measures, as represented by overlapping confidence intervals (CIs) [21]. The only exception was for exercise oscillatory ventilation, which had lower sensitivity. SROC AUC values were similar for all CRF measures, although CIs were not reported for three of the four estimates. All dOR and SROC AUC values suggest CRF had high prognostic accuracy to predict severe cardiovascular events. When directly compared in a sub-group of nine studies, $\dot{V}$_E_/$\dot{V}$CO_2_ slope and $\dot{V}$O_2peak_ had similar prognostic capabilities (dOR 4.8 [95% CI 3.6–6.5] for $\dot{V}$_E_/$\dot{V}$CO_2_ slope; dOR 4.5 [95% CI 3.1–6.4] for $\dot{V}$O_2peak_). Exercise oscillatory ventilation had higher prognostic accuracy for future severe cardiovascular events (*n* = 2 studies; dOR 6.4 [95% CI 3.5–11.9]) than $\dot{V}$_E_/$\dot{V}$CO_2_ slope and $\dot{V}$O_2peak_.

#### Diagnostic Accuracy Among Children and Adolescents

##### Cardiovascular Disease Risk

There was low certainty, from a single review, for $\dot{V}$O_2max_ to detect cardiovascular disease risk among children and adolescents [23]. This meta-analysis stratified summary statistics by sex, with larger dOR values observed for males compared with females. Sensitivity, specificity, and SROC AUC values were similar for both sexes, with overlapping CIs. $\dot{V}$O_2max_ had moderate (females) to high (males) diagnostic accuracy to detect cardiovascular disease risk.

#### Diagnostic Accuracy Among Adults

##### Coronary Artery Disease

There was very low certainty, from three reviews, for CRF to detect coronary artery disease in chronic disease populations [22, 24,25]. Of these, only two reported dOR, SROC AUC, sensitivity, or specificity values [22, 24]. The results showed how dOR, sensitivity, and SROC AUC were higher for treadmill exercise performance in females with suspected and subclinical coronary artery disease [24] compared to treadmill/cycle ergometry exercise performance in males and females with type 2 diabetes [22], but specificity was similar. Based on dOR and SROC AUC values, the diagnostic accuracy of CRF was high in females with suspected and subclinical coronary artery disease [24] and low-to-moderate in males and females with type 2 diabetes [22]. A sub-analysis by Dun et al. [22] compared whether type of exercise (treadmill vs. cycle ergometry) influenced sensitivity and specificity. There was no significant difference observed between the two types of exercise for both sensitivity and specificity (sensitivity 0.56 [95% CI 0.37–0.74] for cycle ergometry and 0.61 [95% CI 0.41–0.80] for treadmill, *p* = 0.69; specificity 0.65 [95% CI 0.55–0.75] for cycle ergometry and 0.63 [95% CI 0.38–0.85] for treadmill, *p* = 0.87) [22].

One meta-analysis by Banerjee et al. [25] presented only hierarchical summary receiver operating characteristic curves, and positive and negative likelihood ratios (LR + , LR -) [25]. The findings from this meta-analysis highlight that among adults without known disease, exercise testing was more useful for confirming coronary artery disease than excluding it. This study suggested that a positive exercise test result (i.e. the participant experienced heart problems) improved the ability to discriminate coronary artery disease in patients aged < 60 years (LR +  = 4.74, 95% CI 3.79–5.93) compared with patients aged ≥ 60 years (LR +  = 2.80, 95% CI 1.77–4.43), in males (LR +  = 4.36, 95% CI 2.89–6.56) compared with females (LR +  = 2.75, 95% CI 1.42–8.92), and from cycle ergometry echocardiography (LR +  = 11.34, 95% CI 6.31–20.38) compared with other exercise test modalities (treadmill walking ECG: LR +  = 3.57, 95% CI 2.71–4.71; treadmill walking echocardiography: LR +  = 7.94, 95% CI 4.67–13.48; cycle ergometry ECG: LR +  = 2.94, 95% CI 2.18–3.96; pharmacological stress testing: LR +  = 6.14, 95% CI 4.27–8.82) [25]. Collectively, these results indicate the diagnostic accuracy of exercise testing depends on the age, sex, and clinical characteristics of participants, the prevalence of the health outcome of interest, and the exercise test modality [25].

##### Mitochondrial Disorders

There was high certainty of evidence for the diagnostic accuracy of absolute load (independent of the participant’s CRF level) and relative load (dependent on the participant’s CRF level) lactate stress tests to detect mitochondrial disorders among adults, according to one meta-analysis [26]. Whilst specificity was similar for both protocols, the absolute load lactate stress test showed higher sensitivity and dOR values, whereas the relative load lactate stress test had higher AUC values. Because the AUC reflects overall diagnostic performance by accounting for the trade‑off between sensitivity and specificity, the relative load lactate stress test may indicate better *overall* discriminative performance. However, when prioritising sensitivity, the absolute load protocol may be preferable. Collectively, both the absolute and relative load lactate stress tests represent valid diagnostic tools for detecting mitochondrial disorders. Sub-group analyses suggested variations lactate cut-off values across studies were likely the primary source of heterogeneity for the relative load lactate stress test protocol.

## Discussion

This overview of systematic reviews with meta-analyses suggests that CRF has moderate-to-high diagnostic accuracy to detect cardiovascular risk among apparently healthy children and cardiovascular disease and mitochondrial disorders among adults with chronic conditions, and high prognostic accuracy to predict cardiovascular events among adults with heart failure. However, the certainty of evidence ranged from low to high certainty due to methodological limitations in the summarised reviews. CRF is recognised as a marker of cardiovascular health [5] and recommended for clinical practice [3, 8], with the American Heart Association advising that CRF should be routinely measured to optimise the assessment of cardiovascular disease risk and better identify individuals at increased risk of poor health [3]. CRF testing in clinical settings is becoming increasingly popular for measuring functional capacity, diagnostic risk stratification, and formulating tailored personalised exercise training programmes [2]. However, CRF is not routinely measured and monitored or assessed in primary or secondary care settings, and as such, the diagnostic accuracy of CRF is potentially underutilised. The findings of this overview support measurement of CRF as a relatively inexpensive, non-invasive, and easily implemented tool to identify groups among populations at risk of adverse cardiovascular events and mitochondrial disorders.

The present overview highlights the diagnostic accuracy of CRF measurements using different test protocols (i.e. submaximal and maximal intensity exercise tests conducted in laboratory and field settings, absolute vs. relative load lactate stress tests) for detecting cardiovascular health outcomes and mitochondrial disorders and examines its prognostic accuracy for predicting cardiovascular events. Evidence from the single prognostic accuracy study indicated that ventilatory efficiency ($\dot{V}$_E_/$\dot{V}$CO_2_ slope, $\dot{V}$O_2_ efficiency slope) and fluctuation (exercise oscillatory ventilation) had relatively greater prognostic accuracy than $\dot{V}$O_2peak_ for predicting severe cardiovascular events among patients with heart failure [21]. Though these measures require indirect calorimetry, ventilatory efficiency and fluctuation measures can be objectively assessed from progressive submaximal aerobic exercise testing. In the context of risk stratification and longer-term management planning, these results highlight both prognostic and practical benefits in selecting these measures over, or alongside, $\dot{V}$O_2peak_, which requires maximal exertion. Regarding diagnostic accuracy, the meta-analysis [26] of mitochondrial disorders highlighted the complexity of recommending a single lactate stress test protocol, especially in the absence of clinically meaningful differences in diagnostic accuracy between absolute and relative load protocols. The authors highlighted how each test protocol had strengths and limitations, with decision making driven by access to equipment and the CRF (i.e. $\dot{V}$O_2max_) and motor skills of individuals [26]. For example, benefits of the relative load lactate stress test include individualised workloads to better control exercise intensity and improve the ability to compare results among patients as all patients would be experiencing a similar level of physiological stress [26]. Our findings highlight potential flexibility in selecting the most appropriate aspect of CRF to assess in different settings. Such decisions are context and population specific, and dependent on test feasibility and clinician discretion.

It is important that the specific clinical presentation and practice context be considered when selecting an objective measure of CRF [28]. In some circumstances, maximal exercise testing may be preferred as it provides valuable information about the reason for test termination, an individual’s perception of effort, and other exercise intolerance metrics (e.g. symptoms such as onset of angina, dyspnoea, and intermittent claudication). Such information can also be used to inform a safe and effective exercise prescription. Alternatively, submaximal testing provides a sufficient exercise stimulus to identify functional and physiological thresholds that can also be used to guide individualised exercise prescription. Healthcare providers and medical professionals may opt for submaximal testing due to participant preference or when maximal testing is contraindicated [29]. Our findings highlight the need for consideration of the feasibility–accuracy trade-off when selecting an appropriate CRF assessment. Our findings also showed no differences in the diagnostic accuracy between treadmill and cycle ergometry, providing flexibility in test selection.

When selecting CRF assessments, diagnostic accuracy metrics should be interpreted alongside practical considerations such as feasibility, resource requirements, patient burden, and intended clinical use. A CRF measure with strong diagnostic performance may be impractical for routine use if it requires specialised equipment or maximal exertion. Conversely, more accessible CRF tests may be appropriate for broader screening despite lower diagnostic accuracy. A test with high sensitivity (and a low LR −) may be particularly useful for ruling out disease, whereas a test with high specificity (and a high LR +) may better support ruling in disease. Although AUC and dOR reflect overall discriminative ability, they do not alone determine rule-in or rule-out utility. Further, CRF can be used as a risk stratification tool to identify adults who may be at increased risk and warrant further clinical assessment [30]. In this regard, prioritising sensitivity, and thus capturing most adults at increased risk, enhances clinical utility and may facilitate early identification and more efficient diagnostic resource allocation. Aligning diagnostic performance with feasibility and clinical context facilitates more informed selection of CRF measures, with diagnostic metrics intended to support, rather than replace, clinical judgment. The findings of this overview support the integration of CRF assessment into practice, particularly for prognostic stratification in heart failure and diagnostic evaluation in suspected mitochondrial disorders (given the higher diagnostic accuracy for these outcomes), while its role in type 2 diabetes, suspected coronary artery disease, and paediatric populations should be interpreted cautiously given lower certainty of evidence. Overall, CRF appears to be a clinically meaningful tool of diagnostic and prognostic value, warranting routine consideration alongside established risk assessments.

The advantages of assessing CRF were emphasised by Sun et al. [24], who summarised the diagnostic accuracy of CRF versus coronary angiography, the ‘gold standard’ measure used to detect coronary artery disease. Coronary angiography is invasive, costly, and associated with adverse events and side effects [24], which highlights the need to develop more appropriate diagnostic tools. Among adults, CRF was shown to have high diagnostic accuracy to detect coronary artery disease and high prognostic accuracy to predict cardiovascular events, albeit with low to moderate certainty, providing further support for CRF as a non-invasive, non-irradiated, well-tolerated, and feasible diagnostic tool. Furthermore, by showcasing the high diagnostic accuracy of lactate stress tests to detect mitochondrial disorders with high certainty, El Guessabi et al. [26] provided additional insight into the benefits of CRF assessment over common diagnostic tools like muscle biopsies, which are invasive and challenging procedures [26].

Interestingly, the diagnostic accuracy and certainty of evidence for CRF differed between coronary artery disease among individuals with chronic disease (low certainty) and mitochondrial disorders (high certainty), likely reflecting several factors. As impaired oxidative phosphorylation is central to mitochondrial disorders, CRF and exercise-related metabolic responses (e.g. lactate accumulation) are closely linked to disease pathophysiology. In contrast, coronary artery disease in populations with chronic conditions (e.g. type 2 diabetes mellitus) is multifactorial, heterogeneous in presentation, and influenced by comorbidities and medications that may attenuate the discriminatory value of CRF. Reference standards also differ, with mitochondrial disorders typically confirmed using specific genetic or histological criteria, whereas coronary artery disease diagnostic approaches vary across studies and may introduce misclassification. Furthermore, the evidence base for mitochondrial disorders was more consistent in design and demonstrated larger effect sizes, supporting higher certainty ratings, whereas studies of coronary artery disease in chronic disease populations were more heterogeneous in methodology and outcome definitions, contributing to lower certainty of evidence.

Assessing multiple aspects of CRF may provide a more comprehensive and potentially more accurate approach to identify those at risk of cardiovascular disease. Additional research is required to determine whether different aspects of CRF are complementary, if there is an optimal combination that enhances health-related diagnostic accuracy, and if so, develop a cardio health fitness test battery. Ideally, multiple aspects of CRF should be extracted from a single test to minimise additional time and resource burden associated with extra testing. For example, $\dot{V}$_E_/$\dot{V}$CO_2_ and $\dot{V}$O_2max_ can both be derived from a progressive aerobic exercise test using indirect calorimetry. As $\dot{V}$_E_/$\dot{V}$CO_2_ does not require maximal exercise intensity, whereas $\dot{V}$O_2max_ does, it would be beneficial to determine if the diagnostic accuracy improves by adding $\dot{V}$O_2max_. If not, then submaximal intensity exercise testing may be preferred from a diagnostic accuracy perspective in clinical settings.

There are limitations of this overview that must be acknowledged. Firstly, owing to the methodological and demographic differences between a small number of reviews, we were limited to qualitatively synthesising the available evidence. We acknowledge that diagnostic accuracy (the ability to correctly identify the presence or absence of current disease) and prognostic accuracy (the ability to predict future health outcomes) represents distinct concepts and their clinical application differ. Our overview primarily focuses on diagnostic accuracy. Prognostic findings were interpreted separately and were not used to inform conclusions regarding current disease detection. Findings were summarised with certainty of evidence ranging from very low to high. Only one of the included reviews had a target population of apparently healthy children and adolescents, with the other reviews examining adults with pre-existing chronic conditions or adults with signs/symptoms or increased risk for coronary artery disease or mitochondrial disorders, and all but one review examined cardiovascular health outcomes. Therefore, additional high-quality age-specific diagnostic evidence for CRF is required to better understand the health-related diagnostic accuracy of CRF in the general population and to determine the prognostic accuracy of CRF to predict outcomes beyond those related to cardiovascular health or mitochondrial disorders, such as all-cause mortality and metabolic disease [31]. Furthermore, as only one included review examined ventilatory fluctuation (exercise oscillatory ventilation) and efficiency (as $\dot{V}$O_2_ efficiency slope), additional research is required to better understand their diagnostic accuracy in comparison with other more widely used CRF measures (e.g. $\dot{V}$O_2max_ or $\dot{V}$O_2peak_) and the degree to which the diagnostic accuracy varies in other clinical populations. We acknowledge that different CRF measures reflect complementary rather than interchangeable dimensions of fitness and underlying disease processes, and that this heterogeneity influences interpretation and clinical application. Our review did not seek to impose a unified physiological model. Instead, CRF was conceptualised as an umbrella construct, reflecting its common operationalisation in clinical and epidemiological research while acknowledging that its component measures are not mechanistically equivalent. Lastly, all included reviews were graded as having low to critically low quality, which was largely due to flaws relating to lack of adjustment for confounding. Strengths of this overview include carefully assessing the review quality and determining the certainty of the evidence. By collating and summarising the available evidence, we identified gaps in understanding that should be prioritised in future research to better advocate for the diagnostic utility of CRF in assessing cardiovascular health and detecting mitochondrial disorders. Specifically, there is a need for higher quality reporting of systematic reviews exploring both maximal and submaximal exercise test protocols, different CRF metrics, other health outcomes, and different population groups.

## Conclusion

CRF has moderate-to-high diagnostic accuracy for detecting cardiovascular risk among apparently healthy children and cardiovascular disease and mitochondrial disorders among adults with chronic conditions, and high prognostic accuracy for predicting cardiovascular events among adults with heart failure, with certainty of evidence ranging from low to high certainty. Despite these promising findings, the overall evidence is tempered by low to critically low methodological quality across the included reviews. Given the strength of CRF as a predictor of health outcomes along with the recent emphasis on the application of CRF in clinical practice [3, 32], the current results add to a growing body of literature that provides an impetus for healthcare providers to promote physical activity both for their patients and the public. The current overview also highlights gaps in current understanding and emphasises the need for further original research and high-quality systematic reviews with meta-analyses to examine the health-related diagnostic accuracy of CRF in the general population across a variety of health outcomes and age groups. Addressing these knowledge gaps will provide a better understanding of the certainty of evidence for how routine CRF testing could be promoted to identify groups at risk for poor health outcomes.

## Supplementary Information

Below is the link to the Electronic Supplementary Material.

Supplementary file1 (DOCX 142 KB)

## Declarations

### Availability of data, code, and other materials

All materials from this study have been included as Electronic Supplementary Material. Additional materials are available upon request.
