# Supplementary material for: Diagnostic and Prognostic Accuracy of Cardiorespiratory Fitness for Detecting Cardiovascular Risk and Mitochondrial Disorders: An Overview of Systematic Reviews with Meta-Analyses
Source: Sports Med. 2026 Aug 4;56(12):3125–39. doi: 10.1007/s40279-026-02503-7 (PMC13759579; doi:10.1007/s40279-026-02503-7)
Supplement: Supplementary file 1 [file 40279_2026_2503_MOESM1_ESM.docx]

**Title**

**Diagnostic and prognostic accuracy of cardiorespiratory fitness for detecting cardiovascular risk and mitochondrial disorders: An overview of systematic reviews with meta-analyses**
Running title: Overview of the health-related diagnostic and prognostic accuracy of cardiorespiratory fitness

**Authors**

Brooklyn J. Fraser *PhD*^1,2^ ORCID: 0000-0002-1752-5431

Bethany Gower *MRes*^2^ ORCID: 0000-0001-6969-6913

Oliver Stanesby *MSc(Epi)*^1, 3^ ORCID: 0000-0002-5996-5881

Costan G. Magnussen *PhD*^2,4,5^ ORCID: 0000-0002-6238-5730

Cristina Cadenas-Sanchez *PhD*^6,7,8,9^ ORCID: 0000-0002-4513-9108

Jean-Philippe Chaput *PhD*^10,11^ ORCID: 0000-0002-5607-5736

Taru Manyanga *PhD*^10,12^ ORCID: 0000-0001-5461-5981

Ryan McGrath *PhD*^2,13,14,15,16^ ORCID: 0000-0002-0644-5524

Katherine Merucci *MLIS*^17^ ORCID: 0000-0003-1981-7013

Jonathan Myers *PhD*^7,8^ ORCID: 0000-0003-2592-136X

Francisco B. Ortega *PhD*^6,9,18^ ORCID: 0000-0003-2001-1121

Ben Singh *PhD*^2^ ORCID: 0000-0002-7227-2406

Stephanie A. Prince *PhD*^19,20^ ORCID: 0000-0001-6729-5649

Justin J. Lang *PhD*^2,10,19,20^ 0000-0002-1768-319X

Grant R. Tomkinson *PhD*^2^ ORCID: 0000-0001-7601-9670

**Affiliations**

1 Menzies Institute for Medical Research, University of Tasmania, Hobart, Tasmania, Australia
2 Alliance for Research in Exercise, Nutrition and Activity (ARENA), School of Allied Health and Human Performance, Adelaide University, Adelaide, South Australia, Australia
3 Baker Heart and Diabetes Institute, Melbourne, Australia
4 Research Centre of Applied and Preventive Cardiovascular Medicine, University of Turku, Turku, Finland
5 Centre for Population Health Research, University of Turku and Turku University Hospital, Turku, Finland
6 Department of Physical Education and Sports, Faculty of Sport Sciences, Sport and Health University Research Institute (iMUDS), University of Granada, Granada, Spain
7 Department of Cardiology, Stanford University, Stanford, California, USA
8 Veterans Affairs Palo Alto Health Care System, Palo Alto, California, USA
9 Centro de Investigación Biomédica en Red Fisiopatología de la Obesidad y Nutrición (CIBERobn), Instituto de Salud Carlos III, Granada, Spain
10 Children’s Hospital of Eastern Ontario Research Institute, Ottawa, Ontario, Canada
11 Department of Pediatrics, Faculty of Medicine, University of Ottawa, Ottawa, Ontario, Canada
12 Division of Medical Sciences, University of Northern British Columbia, Prince George, BC, Canada
13 Healthy Aging North Dakota (HAND), North Dakota State University, Fargo, ND, USA.
14 Department of Health, Nutrition, and Exercise Sciences, North Dakota State University, Fargo, ND, USA
15 Fargo VA Healthcare System, Fargo, ND, USA
16 Department of Geriatrics, University of North Dakota, Grand Forks, ND, USA
17 Health Library, Health Canada, Ottawa, Ontario, Canada
18 Faculty of Sport and Health Sciences, University of Jyväskylä, Jyväskylä, Finland
19 Centre for Surveillance and Applied Research, Public Health Agency of Canada, Ottawa, Ontario, Canada
20 School of Epidemiology and Public Health, Faculty of Medicine, University of Ottawa, Ottawa, Ontario, Canada

**Corresponding author**

Brooklyn J Fraser

Menzies Institute for Medical Research

University of Tasmania

Private Bag 23

Hobart 7001

Tasmania

Australia

Phone: +61 3 6226 7700

E-mail: [brooklyn.fraser@utas.edu.au](mailto:brooklyn.fraser@utas.edu.au)

**Online supplement**

**eAppendix 1.** PRIOR Checklist.

**(Gates M, Gates A, Pieper D, et al. Reporting guideline for overviews of reviews of healthcare interventions: development of the PRIOR statement. *BMJ* 2022;378:e070849. doi:10.1136/bmj-2022-070849.)**

| **Section**  Topic | **#** | **Item** | **Location reported** |
| --- | --- | --- | --- |
| **TITLE** | | |  |
| Title | 1 | Identify the report as an overview of reviews. | Page 1 – cover page |
| **ABSTRACT** | | |  |
| Abstract | 2 | Provide a comprehensive and accurate summary of the purpose, methods, and results of the overview of reviews. | Page 3 - 4 |
| **INTRODUCTION** | | |  |
| Rationale | 3 | Describe the rationale for conducting the overview of reviews in the context of existing knowledge. | Page 5 - 6 |
| Objectives | 4 | Provide an explicit statement of the objective(s) or question(s) addressed by the overview of reviews. | Page 6 |
| **METHODS** | | |  |
| Eligibility criteria | 5a | Specify the inclusion and exclusion criteria for the overview of reviews. If supplemental primary studies were included, this should be stated, with a rationale. | Page 7 - 8 |
|  | 5b | Specify the definition of ‘systematic review’ as used in the inclusion criteria for the overview of reviews. | Page 7 |
| Information sources | 6 | Specify all databases, registers, websites, organizations, reference lists, and other sources searched or consulted to identify systematic reviews and supplemental primary studies (if included).  Specify the date when each source was last searched or consulted. | Page 8 |
| Search strategy | 7 | Present the full search strategies for all databases, registers and websites, such that they could be reproduced. Describe any search filters and limits applied. | eAppendix 2 |
| Selection process | 8a | Describe the methods used to decide whether a systematic review or supplemental primary study (if included) met the inclusion criteria of the overview of reviews. | Page 8 - 9 |
|  | 8b | Describe how overlap in the populations, interventions, comparators, and/or outcomes of systematic reviews was identified and managed during study selection. | Page 9 |
| Data collection process | 9a | Describe the methods used to collect data from reports. | Page 9 |
|  | 9b | If applicable, describe the methods used to identify and manage primary study overlap at the level of the comparison and outcome during data collection. For each outcome, specify the method used to illustrate and/or quantify the degree of primary study overlap across systematic reviews. | Page 10 |
|  | 9c | If applicable, specify the methods used to manage discrepant data across systematic reviews during data collection. | NA |
| Data items | 10 | List and define all variables and outcomes for which data were sought. Describe any assumptions made and/or measures taken to identify and clarify missing or unclear information. | Page 9 |
| Risk of bias assessment | 11a | Describe the methods used to *assess* risk of bias or methodological quality of the included systematic reviews. | Page 9 |
|  | 11b | Describe the methods used to *collect* data on (from the systematic reviews) and/or *assess* the risk of bias of the primary studies included in the systematic reviews. Provide a justification for instances where flawed, incomplete, or missing assessments are identified but not re-assessed. | Page 9 |
|  | 11c | Describe the methods used to *assess* the risk of bias of supplemental primary studies (if included). | Page 9 |
| Synthesis methods | 12a | Describe the methods used to summarize or synthesize results and provide a rationale for the choice(s). | Page 9 - 10 |
|  | 12b | Describe any methods used to explore possible causes of heterogeneity among results. | Page 9 - 10 |
|  | 12c | Describe any sensitivity analyses conducted to assess the robustness of the synthesized results. | NA |
| Reporting bias assessment | 13 | Describe the methods used to *collect* data on (from the systematic reviews) and/or *assess* the risk of bias due to missing results in a summary or synthesis (arising from reporting biases at the levels of the systematic reviews, primary studies, and supplemental primary studies, if included). | Page 9 |
| Certainty assessment | 14 | Describe the methods used to *collect* data on (from the systematic reviews) and/or *assess* certainty (or confidence) in the body of evidence for an outcome. | Page 9 |
| **RESULTS** | | |  |
| Systematic review and supplemental primary study selection | 15a | Describe the results of the search and selection process, including the number of records screened, assessed for eligibility, and included in the overview of reviews, ideally with a flow diagram. | Page 11, and Figure 1 |
|  | 15b | Provide a list of studies that might appear to meet the inclusion criteria, but were excluded, with the main reason for exclusion. | eAppendix 3 |
| Characteristics of systematic reviews and supplemental primary studies | 16 | Cite each included systematic review and supplemental primary study (if included) and present its characteristics. | Page 11 - 12 and Table 1 |
| Primary study overlap | 17 | Describe the extent of primary study overlap across the included systematic reviews. | Page 11 |
| Risk of bias in systematic reviews, primary studies, and  supplemental primary studies | 18a | Present assessments of risk of bias or methodological quality for each included systematic review. | Page 13, Table 1 |
|  | 18b | Present assessments (*collected* from systematic reviews or *assessed* anew) of the risk of bias of the primary studies included in the systematic reviews. | Page 13 |
|  | 18c | Present assessments of the risk of bias of supplemental primary studies (if included). | Page 13 |
| Summary or synthesis of results | 19a | For all outcomes, summarize the evidence from the systematic reviews and supplemental primary studies (if included). If meta-analyses were done, present for each the summary estimate and its precision and measures of statistical heterogeneity. If comparing groups, describe the direction of the effect. | Figure 2, 3, 4, and 5 |
|  | 19b | If meta-analyses were done, present results of all investigations of possible causes of heterogeneity. | NA |
|  | 19c | If meta-analyses were done, present results of all sensitivity analyses conducted to assess the robustness of synthesized results. | NA |
| Reporting biases | 20 | Present assessments (*collected* from systematic reviews and/or *assessed* anew) of the risk of bias due to missing primary studies, analyses, or results in a summary or synthesis (arising from reporting biases at the levels of the systematic reviews, primary studies, and supplemental primary studies, if included) for each summary or synthesis assessed. | Page 13 - 16 |
| Certainty of evidence | 21 | Present assessments (*collected* or *assessed* anew) of certainty (or confidence) in the body of evidence for each outcome. | Page 13 - 16 and eAppendix 4 |
| **DISCUSSION** | | |  |
| Discussion | 22a | Summarize the main findings, including any discrepancies in findings across the included systematic reviews and supplemental primary studies (if included). | Page 16 |
|  | 22b | Provide a general interpretation of the results in the context of other evidence. | Page 16 - 23 |
|  | 22c | Discuss any limitations of the evidence from systematic reviews, their primary studies, and supplemental primary studies (if included) included in the overview of reviews. Discuss any limitations of the overview of reviews methods used. | Page 19 - 23 |
|  | 22d | Discuss implications for practice, policy, and future research (both systematic reviews and primary research). Consider the relevance of the findings to the end users of the overview of reviews, e.g., healthcare providers, policymakers, patients, among others. | Page 16 - 23 |
| **OTHER INFORMATION** | | |  |
| Registration and protocol | 23a | Provide registration information for the overview of reviews, including register name and registration number, or state that the overview of reviews was not registered. | Page 4 and 6 |
|  | 23b | Indicate where the overview of reviews protocol can be accessed, or state that a protocol was not prepared. | Page 4 and 6 |
|  | 23c | Describe and explain any amendments to information provided at registration or in the protocol. Indicate the stage of the overview of reviews at which amendments were made. | Page 11 |
| Support | 24 | Describe sources of financial or non-financial support for the overview of reviews, and the role of the funders or sponsors in the overview of reviews. | Page 24 |
| Competing interests | 25 | Declare any competing interests of the overview of reviews' authors. | Page 24 |
| Author information | 26a | Provide contact information for the corresponding author. | Page 2 |
|  | 26b | Describe the contributions of individual authors and identify the guarantor of the overview of reviews. | Page 24 - 25 |
| Availability of data and other materials | 27 | Report which of the following are available, where they can be found, and under which conditions they may be accessed: template data collection forms; data collected from included systematic reviews and supplemental primary studies; analytic code; any other materials used in the overview of reviews. | Page 24 |

**eAppendix 2.** Search strategy.

**MEDLINE via OVID:** 1946 to 18 November 2022, then 1 November to 8 March 2024, and then 1 February 2024 to 11 April 2025.

| **#** | **Searches** |
| --- | --- |
| 1 | *Cardiorespiratory fitness/ |
| 2 | ((cardiorespirator* or cardio respirator* or aerobic or cardiopulmonar* or cardio pulmonar* or cardiovascular* or cardio vascular* or physical* work* or cardiometaboli* or cardio metaboli*) adj4 (fitness or capacit* or endurance or perform* or health*)).ti,kf. or ((cardiorespirator* or cardio respirator* or aerobic or cardiopulmonar* or cardio pulmonar* or cardiovascular* or cardio vascular* or physical* work* or cardiometaboli* or cardio metaboli*) adj4 (fitness or capacit* or endurance or perform* or health*)).ab. /freq=2 |
| 3 | (Cardiorespiratory fitness and CRF).ab. |
| 4 | (Cardiovascular Health Study and CHS).ab. |
| 5 | exp *exercise tests/ |
| 6 | ((minute or mile or distance or timed) adj3 run).ti,kf. or ((minute or mile or distance or timed) adj3 run).ab. /freq=2 |
| 7 | ((fitness or exercise or endurance or step or walk or run or beep or tread?mill or ergometry or eurofit or stress) adj3 test*).ti,kf. or ((fitness or exercise or endurance or step or walk or run or beep or tread?mill or ergometry or eurofit or stress) adj3 test*).ab. /freq=2 |
| 8 | ((functional or aerobic or exercise) adj3 (capacity or endurance)).ti,kf. or ((functional or aerobic or exercise) adj3 (capacity or endurance)).ab. /freq=2 |
| 9 | ((Submaximal or maximal or graded) adj3 (treadmill? or tread mill? or ergometer?)).ti,ab,kf. |
| 10 | (maxim* oxygen or peak oxygen or VO2*).ti,kf. or (maxim* oxygen or peak oxygen or VO2*).ab. /freq=2 |
| 11 | or/1-10 [CARDIORESPIRATORY FITNESS] |
| 12 | (meta-analysis or systematic review).pt. |
| 13 | meta-analysis/ or systematic review/ or meta-analysis as topic/ or systematic review as topic/ |
| 14 | ((systematic* adj3 (review* or overview*)) or (methodologic* adj3 (review* or overview*))).ti,ab,kf. |
| 15 | (cochrane review* or ((umbrella* or mapping or integrative or integrated) adj3 (review* or overview*))).ti,ab,kf. |
| 16 | ((quantitative adj3 (review* or overview* or synthes*)) or (research adj3 (integrati* or overview* or synthes*))).ti,ab,kf. |
| 17 | "review of reviews".ti,ab,kf,kw. |
| 18 | (meta analy* or metanaly* or meta synthes* or metasynthes* or meta ethnography).ti,ab,kf,kw. |
| 19 | (cochrane or evidence report).jw. |
| 20 | ((search* or medline or pubmed or embase or Cochrane or scopus or "web of science" or "sources of information" or data sources or following databases) and (study selection or selection criteria or eligibility criteria or inclusion criteria or exclusion criteria)).ti,ab. |
| 21 | (systematic literature adj (research or search)).ti. |
| 22 | or/12-21 [SYSTEMATIC REVIEWS + META ANALYSES] |
| 23 | (letter or editorial or comment or case reports or historical article or report or protocol or protocols or retraction of publication or retracted publication or published erratum).pt. or retraction of publication as topic/ or reply.ti. |
| 24 | 22 not 23 [adapted from "SR / MA / HTA / ITC - MEDLINE, Embase, PsycInfo. In: CADTH Search Filters Database. Ottawa: CADTH; 2022: https://searchfilters.cadth.ca/link/33." Accessed 2022-10-28. and "Salvador-Oliván JA, Marco-Cuenca G, Arquero-Avilés R. Development of an efficient search filter to retrieve systematic reviews from PubMed. J Med Libr Assoc. 2021 Oct 1;109(4):561-574. doi: 10.5195/jmla.2021.1223."] |
| 25 | 11 and 24 [CRF + SYSTEMATIC REVIEWS + META ANALYSES] |
| 26 | (exp animals/ or exp animal experimentation/ or exp models, animal/) not (humans/ or exp human experimentation/ or exp persons/ or human*.ti.) |
| 27 | (animal* or ape or apes or chimpanzee* or gerbil* or guineapig* or guinea pig* or hamster? or hare or hares or macaque* or mammal* or mice or monkey* or mouse or primate* or rabbit* or rat or rats or rodent?).ti. |
| 28 | 26 or 27 [ANIMAL STUDIES] |
| 29 | 25 not 28 |
| 30 | limit 29 to yr="2002-current" |

**Embase via OVID:** 1974 to 18 November 2022, then 1 November to 8 March 2024, and then 1 February 2024 to 11 April 2025.

| **#** | **Searches** |
| --- | --- |
| 1 | *Cardiorespiratory fitness/ |
| 2 | ((cardiorespirator* or cardio respirator* or aerobic or cardiopulmonar* or cardio pulmonar* or cardiovascular* or cardio vascular* or physical* work* or cardiometaboli* or cardio metaboli*) adj4 (fitness or capacit* or endurance or perform* or health*)).ti,kf. or ((cardiorespirator* or cardio respirator* or aerobic or cardiopulmonar* or cardio pulmonar* or cardiovascular* or cardio vascular* or physical* work* or cardiometaboli* or cardio metaboli*) adj4 (fitness or capacit* or endurance or perform* or health*)).ab. /freq=2 |
| 3 | (Cardiorespiratory fitness and CRF).ab. |
| 4 | (Cardiovascular Health Study and CHS).ab. |
| 5 | exp *exercise test/ |
| 6 | ((minute or mile or distance or timed) adj3 run).ti,kf. or ((minute or mile or distance or timed) adj3 run).ab. /freq=2 |
| 7 | ((fitness or exercise or endurance or step or walk or run or beep or tread?mill or ergometry or eurofit or stress) adj3 test*).ti,kf. or ((fitness or exercise or endurance or step or walk or run or beep or tread?mill or ergometry or eurofit or stress) adj3 test*).ab. /freq=2 |
| 8 | ((functional or aerobic or exercise) adj3 (capacity or endurance)).ti,kf. or ((functional or aerobic or exercise) adj3 (capacity or endurance)).ab. /freq=2 |
| 9 | ((Submaximal or maximal or graded) adj3 (treadmill? or tread mill? or ergometer?)).ti,ab,kf. |
| 10 | (maxim* oxygen or peak oxygen or VO2*).ti,kf,kw. or (maxim* oxygen or peak oxygen or VO2*).ab. /freq=2 |
| 11 | or/1-10 [CARDIORESPIRATORY FITNESS] |
| 12 | meta-analysis/ or systematic review/ or "meta analysis (topic)"/ or "systematic review (topic)"/ |
| 13 | ((systematic* adj3 (review* or overview*)) or (methodologic* adj3 (review* or overview*))).ti,ab,kf. |
| 14 | (cochrane review* or ((umbrella* or mapping or integrative or integrated) adj3 (review* or overview*))).ti,ab,kf. |
| 15 | ((quantitative adj3 (review* or overview* or synthes*)) or (research adj3 (integrati* or overview* or synthes*))).ti,ab,kf. |
| 16 | "review of reviews".ti,ab,kf,kw. |
| 17 | (meta analy* or metanaly* or meta synthes* or metasynthes* or meta ethnography).ti,ab,kf,kw. |
| 18 | (cochrane or evidence report).jx. |
| 19 | ((search* or medline or pubmed or embase or Cochrane or scopus or "web of science" or "sources of information" or data sources or following databases) and (study selection or selection criteria or eligibility criteria or inclusion criteria or exclusion criteria)).ti,ab. |
| 20 | (systematic literature adj (research or search)).ti. |
| 21 | or/12-20 [SYSTEMATIC REVIEWS + META ANALYSES] |
| 22 | (letter or editorial or conference abstract or note or short survey or erratum).pt. or reply.ti. |
| 23 | 21 not 22 [adapted from "SR / MA / HTA / ITC - MEDLINE, Embase, PsycInfo. In: CADTH Search Filters Database. Ottawa: CADTH; 2022: https://searchfilters.cadth.ca/link/33." Accessed 2022-10-28. and "Salvador-Oliván JA, Marco-Cuenca G, Arquero-Avilés R. Development of an efficient search filter to retrieve systematic reviews from PubMed. J Med Libr Assoc. 2021 Oct 1;109(4):561-574. doi: 10.5195/jmla.2021.1223."] |
| 24 | 11 and 23 [ CRF + SYSTEMATIC REVIEWS + META ANALYSES] |
| 25 | (animals/ or exp animal experiment/ or exp animal model/) not (humans/ or exp human experiment/ or exp named groups of persons/ or human*.ti.) |
| 26 | (animal* or ape or apes or chimpanzee* or gerbil* or guineapig* or guinea pig* or hamster? or hare or hares or macaque* or mammal* or mice or monkey* or mouse or primate* or rabbit* or rat or rats or rodent?).ti. |
| 27 | 25 or 26 [ANIMAL STUDIES] |
| 28 | 24 not 27 |
| 29 | limit 28 to yr="2002-current" |

**Scopus:**

( ( ( TITLE ( ( cardiorespirator*  OR  "cardio respirator*"  OR  aerobic  OR  cardiopulmonar*  OR  "cardio pulmonar*"  OR  cardiovascular*  OR  "cardio vascular*"  OR  "physical* work*"  OR  cardiometaboli*  OR  "cardio metaboli*" )  W/4  ( fitness  OR  capacit*  OR  endurance  OR  perform*  OR  health* ) )  OR  KEY ( ( cardiorespirator*  OR  "cardio respirator*"  OR  aerobic  OR  cardiopulmonar*  OR  "cardio pulmonar*"  OR  cardiovascular*  OR  "cardio vascular*"  OR  "physical* work*"  OR  cardiometaboli*  OR  "cardio metaboli*" )  W/4  ( fitness  OR  capacit*  OR  endurance  OR  perform*  OR  health* ) )  OR  ABS ( "Cardiorespiratory fitness"  AND  "CRF" )  OR  ABS ( "Cardiovascular Health Study"  AND  "CHS" )  OR  TITLE ( ( minute  OR  mile  OR  distance  OR  timed )  W/3  run )  OR  ABS ( ( minute  OR  mile  OR  distance  OR  timed )  W/3  run )  OR  TITLE ( ( fitness  OR  exercise  OR  endurance  OR  step  OR  walk  OR  run  OR  beep  OR  "tread*mill"  OR  ergometry  OR  eurofit  OR  stress )  W/3  test* )  OR  ABS ( ( fitness  OR  exercise  OR  endurance  OR  step  OR  walk  OR  run  OR  beep  OR  "tread*mill"  OR  ergometry  OR  eurofit  OR  stress )  W/3  test* )  OR  TITLE ( ( functional  OR  aerobic  OR  exercise )  W/3  ( capacity  OR  endurance ) )  OR  KEY ( ( functional  OR  aerobic  OR  exercise )  W/3  ( capacity  OR  endurance ) )  OR  TITLE-ABS-KEY ( ( submaximal  OR  maximal  OR  graded )  W/3  ( treadmill*  OR  "tread mill*"  OR  ergometer* ) )  OR  TITLE ( "maxim* oxygen"  OR  "peak oxygen"  OR  vo2* )  OR  KEY ( "maxim* oxygen"  OR  "peak oxygen"  OR  vo2* ) ) )  AND  ( ( INDEXTERMS ( meta-analysis )  OR  INDEXTERMS ( "systematic review" )  OR  INDEXTERMS ( "meta analysis (topic)" )  OR  INDEXTERMS ( "systematic review (topic)" )  OR  INDEXTERMS ( "meta-analysis as topic" )  OR  INDEXTERMS ( "systematic review as topic" )  OR  TITLE-ABS-KEY ( ( systematic*  W/3  ( review*  OR  overview* ) )  OR  ( methodologic*  W/3  ( review*  OR  overview* ) ) )  OR  TITLE-ABS-KEY ( "cochrane review*"  OR  ( ( umbrella*  OR  mapping  OR  integrative  OR  integrated )  W/3  ( review*  OR  overview* ) ) )  OR  TITLE-ABS-KEY ( ( quantitative  W/3  ( review*  OR  overview*  OR  synthes* ) )  OR  ( research  W/3  ( integrati*  OR  overview*  OR  synthes* ) ) )  OR  TITLE-ABS-KEY ( "review of reviews" )  OR  TITLE-ABS-KEY ( "meta analy*"  OR  metanaly*  OR  "meta synthes*"  OR  metasynthes*  OR  "meta ethnography" )  OR  SRCTITLE ( cochrane  OR  "evidence report" )  OR  TITLE-ABS ( ( search*  OR  medline  OR  pubmed  OR  embase  OR  cochrane  OR  scopus  OR  "web of science"  OR  "sources of information"  OR  "data sources"  OR  "following databases" )  AND  ( "study selection"  OR  "selection criteria"  OR  "eligibility criteria"  OR  "inclusion criteria"  OR  "exclusion criteria" ) )  OR  TITLE ( "systematic literature"  W/1  ( research  OR  search ) ) )  AND NOT  ( SRCTYPE ( p  OR  n  OR  r )  OR  DOCTYPE ( "cp"  OR  "ed"  OR  "er"  OR  "le"  OR  "no"  OR  "rp" )  OR  TITLE ( reply ) ) )  AND  ( PUBYEAR  >  2001 ) )  AND NOT  ( ( INDEXTERMS ( animal  OR  animals  OR  "animal experiment"  OR  "animal experimentation"  OR  "animal model"  OR  "models, animal" )  AND NOT  ( INDEXTERMS ( humans  OR  "human experiment"  OR  "human experimentation"  OR  "named groups of persons"  OR  persons )  OR  TITLE ( human* ) ) ) )

**CINAHL via EBSCOhost:** 1981 to 18 November 2022, then 1 November to 8 March 2024, and then 1 February 2024 to 11 April 2025.

| # | **Query** | **Limiters/Expanders** |
| --- | --- | --- |
| S28 | S22 NOT S27 | Limiters - Published Date: 20020101- |
| S27 | S25 OR S26 |  |
| S26 | TI animal* or ape or apes or chimpanzee* or gerbil* or guineapig* or guinea pig* or hamster# or hare or hares or macaque* or mammal* or mice or monkey* or mouse or primate* or rabbit* or rat or rats or rodent# |  |
| S25 | S23 NOT 24 |  |
| S24 | MH "human" OR TI human* |  |
| S23 | MH "animals+" OR MH "animal studies" OR MH "Models, Biological" |  |
| S22 | S8 AND S21 |  |
| S21 | S19 NOT S20 |  |
| S20 | PT ( letter or editorial or commentary or case study or historical material or protocol or response ) OR MH "Scientific Misconduct" OR TI reply |  |
| S19 | S9 OR S10 OR S11 OR S12 OR S13 OR S14 OR S15 OR S16 OR S17 OR S18 |  |
| S18 | TI (systematic literature N1 (research or search)) |  |
| S17 | TI ( ((search* or medline or pubmed or embase or Cochrane or scopus or "web of science" or "sources of information" or data sources or following databases) and (study selection or selection criteria or eligibility criteria or inclusion criteria or exclusion criteria)) ) OR AB ( ((search* or medline or pubmed or embase or Cochrane or scopus or "web of science" or "sources of information" or data sources or following databases) and (study selection or selection criteria or eligibility criteria or inclusion criteria or exclusion criteria)) ) |  |
| S16 | SO (cochrane or evidence report) |  |
| S15 | TI ( (meta analy* or metanaly* or meta synthes* or metasynthes* or meta ethnography) ) OR AB ( (meta analy* or metanaly* or meta synthes* or metasynthes* or meta ethnography) ) |  |
| S14 | TI "review of reviews" OR AB "review of reviews" |  |
| S13 | TI ( ((quantitative N3 (review* or overview* or synthes*)) or (research N3 (integrati* or overview* or synthes*))) ) OR AB ( ((quantitative N3 (review* or overview* or synthes*)) or (research N3 (integrati* or overview* or synthes*))) ) |  |
| S12 | TI ( (cochrane review* or ((umbrella* or mapping or integrative or integrated) N3 (review* or overview*))) ) OR AB ( (cochrane review* or ((umbrella* or mapping or integrative or integrated) N3 (review* or overview*))) ) |  |
| S11 | TI ( ((systematic* N3 (review* or overview*)) or (methodologic* N3 (review* or overview*))) ) OR AB ( ((systematic* N3 (review* or overview*)) or (methodologic* N3 (review* or overview*))) ) |  |
| S10 | MH "meta analysis" OR MH "systematic review" |  |
| S9 | PT "systematic review" OR PT "meta analysis" |  |
| S8 | S1 OR S2 OR S3 OR S4 OR S5 OR S6 OR S7 |  |
| S7 | TI ( maxim* oxygen or peak oxygen or VO2* ) OR AB ( maxim* oxygen or peak oxygen or VO2* ) |  |
| S6 | TI ( (Submaximal or maximal or graded) N3 (treadmill# or tread mill# or ergometer#) ) OR AB ( (Submaximal or maximal or graded) N3 (treadmill# or tread mill# or ergometer#) ) |  |
| S5 | TI ( (functional or aerobic or exercise) N3 (capacity or endurance) ) OR AB ( (functional or aerobic or exercise) N3 (capacity or endurance) ) |  |
| S4 | TI ( (fitness or exercise or endurance or step or walk or run or beep or tread#mill or ergometry or eurofit or stress) N3 test* ) OR AB ( (fitness or exercise or endurance or step or walk or run or beep or tread#mill or ergometry or eurofit or stress) N3 test* ) |  |
| S3 | TI ( (minute or mile or distance or timed) N3 run ) OR AB ( (minute or mile or distance or timed) N3 run ) |  |
| S2 | TI ( (cardiorespirator* or cardio respirator* or aerobic or cardiopulmonar* or cardio pulmonar* or cardiovascular* or cardio vascular* or physical* work* or cardiometaboli* or cardio metaboli*) N4 (fitness or capacit* or endurance or perform* or health*) ) OR AB ( (cardiorespirator* or cardio respirator* or aerobic or cardiopulmonar* or cardio pulmonar* or cardiovascular* or cardio vascular* or physical* work* or cardiometaboli* or cardio metaboli*) N4 (fitness or capacit* or endurance or perform* or health*) ) |  |
| S1 | (MM "Cardiorespiratory Fitness") or (MM "Exercise Test+") |  |

**SPORTDiscus via EBSCOhost**: 1930 to 18 November 2022, then 1 November to 8 March 2024, and then 1 February 2024 to 11 April 2025.

| **#** | **Query** | **Limiters/Expanders** |
| --- | --- | --- |
| S23 | S21 NOT S22 | Limiters - Published Date: 20020101- |
| S22 | TI animal* or ape or apes or chimpanzee* or gerbil* or guineapig* or guinea pig* or hamster# or hare or hares or macaque* or mammal* or mice or monkey* or mouse or primate* or rabbit* or rat or rats or rodent# |  |
| S21 | S9 AND S20 |  |
| S20 | S18 NOT S19 |  |
| S19 | TI reply |  |
| S18 | S10 OR S11 OR S12 OR S13 OR S14 OR S15 OR S16 OR S17 |  |
| S17 | TI (systematic literature N1 (research or search)) |  |
| S16 | TI ( ((search* or medline or pubmed or embase or Cochrane or scopus or "web of science" or "sources of information" or data sources or following databases) and (study selection or selection criteria or eligibility criteria or inclusion criteria or exclusion criteria)) ) OR AB ( ((search* or medline or pubmed or embase or Cochrane or scopus or "web of science" or "sources of information" or data sources or following databases) and (study selection or selection criteria or eligibility criteria or inclusion criteria or exclusion criteria)) ) |  |
| S15 | TI ( (meta analy* or metanaly* or meta synthes* or metasynthes* or meta ethnography) ) OR AB ( (meta analy* or metanaly* or meta synthes* or metasynthes* or meta ethnography) ) |  |
| S14 | TI "review of reviews" OR AB "review of reviews" |  |
| S13 | TI ( (quantitative N3 (review* or overview* or synthes*)) or (research N3 (integrati* or overview* or synthes*)) ) OR AB ( (quantitative N3 (review* or overview* or synthes*)) or (research N3 (integrati* or overview* or synthes*)) ) |  |
| S12 | TI ( cochrane review* or ((umbrella* or mapping or integrative or integrated) N3 (review* or overview*)) ) OR AB ( cochrane review* or ((umbrella* or mapping or integrative or integrated) N3 (review* or overview*)) ) |  |
| S11 | TI ( (systematic* N3 (review* or overview*)) or (methodologic* N3 (review* or overview*)) ) OR AB ( (systematic* N3 (review* or overview*)) or (methodologic* N3 (review* or overview*)) ) |  |
| S10 | PT review |  |
| S9 | S1 OR S2 OR S3 OR S4 OR S5 OR S6 OR S7 OR S8 |  |
| S8 | TI ( maxim* oxygen or peak oxygen or VO2* ) OR AB ( maxim* oxygen or peak oxygen or VO2* ) |  |
| S7 | TI ( (Submaximal or maximal or graded) N3 (treadmill# or tread mill# or ergometer#) ) OR AB ( (Submaximal or maximal or graded) N3 (treadmill# or tread mill# or ergometer#) ) |  |
| S6 | TI ( (functional or aerobic or exercise) N3 (capacity or endurance) ) OR AB ( (functional or aerobic or exercise) N3 (capacity or endurance) ) |  |
| S5 | TI ( (fitness or exercise or endurance or step or walk or run or beep or tread#mill or ergometry or eurofit or stress) N3 test* ) OR AB ( (fitness or exercise or endurance or step or walk or run or beep or tread#mill or ergometry or eurofit or stress) N3 test* ) |  |
| S4 | TI ( (minute or mile or distance or timed) N3 run ) OR AB ( (minute or mile or distance or timed) N3 run ) TI ( (fitness or exercise or endurance or step or walk or run or beep or tread#mill or ergometry or eurofit or stress) N3 test* ) OR AB ( (fitness or exercise or endurance or step or walk or run or beep or tread#mill or ergometry or eurofit or stress) N3 test* ) |  |
| S3 | DE "EXERCISE tests" OR DE "STRESS echocardiography" OR DE "TREADMILL exercise tests" |  |
| S2 | TI ( (cardiorespirator* or cardio respirator* or aerobic or cardiopulmonar* or cardio pulmonar* or cardiovascular* or cardio vascular* or physical* work* or cardiometaboli* or cardio metaboli*) N4 (fitness or capacit* or endurance or perform* or health*) ) OR AB ( (cardiorespirator* or cardio respirator* or aerobic or cardiopulmonar* or cardio pulmonar* or cardiovascular* or cardio vascular* or physical* work* or cardiometaboli* or cardio metaboli*) N4 (fitness or capacit* or endurance or perform* or health*) ) |  |
| S1 | DE "CARDIOVASCULAR fitness" OR DE "CARDIOPULMONARY fitness" |  |

^†^This search strategy was developed for a large overview of reviews project exploring the associations between CRF and health outcomes across the lifespan.

**eAppendix 3.** Excluded articles (n=218) after full-text review with reasons.

1. Agostinis-Sobrinho, C., Ramirez-Velez, R., Garcia-Hermoso, A., Rosario, R., Moreira, C., Lopes, L., Martinkenas, A., Mota, J., & Santos, R. (2019). The combined association of adherence to Mediterranean diet, muscular and cardiorespiratory fitness on low-grade inflammation in adolescents: a pooled analysis. European Journal of Nutrition, 58(7), 2649-2656.  **Reason: Not a systematic review**
2. Alexandrou, M. E., P. Theodorakopoulou, M., Boutou, A., Pella, E., Boulmpou, A., Papadopoulos, C. E., Zafeiridis, A., Papagianni, A., & Sarafidis, P. (2021). Cardiorespiratory fitness assessed by cardiopulmonary exercise testing between different stages of pre-dialysis chronic kidney disease: A systematic review and meta analysis. Nephrology, 26(12), 972-980.  **Reason: Not focused on diagnostic accuracy of CRF (Adult study)**
3. Al-Huda, F., Shapiro, G. D., Davenport, M. H., Bertagnolli, M., & Dayan, N. (2022). Association between Cardiorespiratory Fitness and Hypertensive Disorders of Pregnancy: A Systematic Review and Meta-Analysis. Journal of Clinical Medicine, 11(15).  **Reason: Not focused on diagnostic accuracy of CRF (Adult study)**
4. Al-Huda, F., Shapiro, G. D., Davenport, M. H., Bertagnolli, M., & Dayan, N. (2022). Association between Cardiorespiratory Fitness and Hypertensive Disorders of Pregnancy: A Systematic Review and Meta-Analysis. Journal of clinical medicine, 11(15), 4364.  **Reason: Not focused on diagnostic accuracy of CRF (Adult study)**
5. Alvares, T. S., de Souza, L. V. M., Soares, R. N., & Lessard, S. J. (2024). Cardiorespiratory fitness is impaired in type 1 and type 2 diabetes: a systematic review, meta-analysis, and meta-regression. Medicine and Science in Sports and Exercise, 56(9), 1553.  **Reason: Not focused on diagnostic accuracy of CRF (Adult study)**
6. Alvarez-Bueno, C., Hillman, C. H., Cavero-Redondo, I., Sanchez-Lopez, M., Pozuelo-Carrascosa, D. P., & Martinez-Vizcaino, V. (2020). Aerobic fitness and academic achievement: A systematic review and meta-analysis. Journal of Sports Sciences, 38(5), 582-589.  **Reason: Wrong outcomes**
7. Alves Donato, A. N., Waclawovsky, A. J., Tonello, L., Firth, J., Smith, L., Stubbs, B., Schuch, F. B., & Boullosa, D. (2021). Association between cardiorespiratory fitness and depressive symptoms in children and adolescents: A systematic review and meta-analysis. Journal of Affective Disorders, 282, 1234-1240.  **Reason: Not focused on diagnostic accuracy of CRF (Child study)**
8. Alves, D. J. F., Bartholomeu-Neto, J., JÚNior, E. R., Zarricueta, B. S. R., NÓBrega, O. T., & CÓRdova, C. (2017). Walking speed, risk factors, and cardiovascular events in older adults--systematic review. Journal of Strength & Conditioning Research, 31(11), 3235-3244.  **Reason: No meta-analysis**
9. Anderson, K. C., Weeldreyer, N. R., Leicht, Z. S., Angadi, S. S., & Liu, Z. (2025). Exercise Intolerance in Type 2 Diabetes: A Systematic Review and Meta‐Analysis. Journal of the American Heart Association, 14(5), e035721.  **Reason: Not focused on diagnostic accuracy of CRF (Adult study)**
10. Andrade Lima, C., Dornelas de Andrade, A., Campos, S. L., Brandao, D. C., Mourato, I. P., & Britto, M. C. A. d. (2018). Six-minute walk test as a determinant of the functional capacity of children and adolescents with cystic fibrosis: A systematic review. Respiratory Medicine, 137, 83-88.  **Reason: No meta-analysis**
11. Andrade, R. D. A., de Menezes, B. A., Vieira, L. L., Daniele, T. M. C., & de Sousa, N. J. F. (2019). Association between physical fitness and cardiovascular risk in young university students: Systematic review. Motricidade, 15(2-3), 75-84.  **Reason: No meta-analysis**
12. Aneni, E. C., Crippa, A., Osondu, C. U., Valero-Elizondo, J., Younus, A., Nasir, K., & Veledar, E. (2017). Estimates of Mortality Benefit From Ideal Cardiovascular Health Metrics: A Dose Response Meta-Analysis. Journal of the American Heart Association, 6(12).  **Reason: No measure of CRF**
13. Arbee-Kalidas, N., Moutlana, H. J., Moodley, Y., Kebalepile, M. M., & Motshabi Chakane, P. (2023). The association between cardiopulmonary exercise testing and postoperative outcomes in patients with lung cancer undergoing lung resection surgery: A systematic review and meta-analysis. PloS one, 18(12), e0295430.  **Reason: Not focused on diagnostic accuracy of CRF (Adult study)**
14. Arena, R., Myers, J., & Guazzi, M. (2008). The clinical and research applications of aerobic capacity and ventilatory efficiency in heart failure: an evidence-based review. Heart Failure Reviews, 13(2), 245-269.  **Reason: No meta-analysis**
15. Aune, D., Norat, T., Leitzmann, M., Tonstad, S., & Vatten, L. J. (2015). Physical activity and the risk of type 2 diabetes: a systematic review and dose-response meta-analysis. European Journal of Epidemiology, 30(7), 529– 542.  **Reason: Not focused on diagnostic accuracy of CRF (Adult study)**
16. Aune, D., Schlesinger, S., Hamer, M., Norat, T., & Riboli, E. (2020) Physical activity and the risk of sudden cardiac death: a systematic review and meta-analysis of prospective studies. BMC Cardiovascular Disorders, 20(1), 318.  **Reason: Not focused on diagnostic accuracy of CRF (Adult study)**
17. Aune, D., Schlesinger, S., Leitzmann, M.F., Tonstad, S., Norat, T., Riboli, E., & Vatten, L.J. (2021) Physical activity and the risk of heart failure: a systematic review and dose-response meta-analysis of prospective studies. European Journal of Epidemiology, 36(4), 367-381.  **Reason: Not focused on diagnostic accuracy of CRF (Adult study)**
18. Badrooj, N., Jayedi, A., & Shab-Bidar, S. (2023). Ideal cardiovascular health metrics and risk of type 2 diabetes: A systematic review and dose-response meta-analysis of prospective cohort studies. Nutrition, metabolism, and cardiovascular diseases : NMCD, 33(11), 2067–2075.  **Reason: No measure of CRF**
19. Baillot, A., Audet, M., Baillargeon, J. P., Dionne, I. J., Valiquette, L., Rosa-Fortin, M. M., Abou Chakra, C. N., Comeau, E., & Langlois, M. F. (2014). Impact of physical activity and fitness in class II and III obese individuals: A systematic review. Obesity Reviews, 15(9), 721-739.  **Reason: No meta-analysis**
20. Barbagelata, L., Masson, W., Bluro, I., Lobo, M., Iglesias, D., & Molinero, G. (2022) Prognostic role of cardiopulmonary exercise testing in pulmonary hypertension: a systematic review and meta-analysis. Advances in Respiratory Medicine, 90(2),109-117.  **Reason: Not focused on diagnostic accuracy of CRF (Adult study)**
21. Barbosa Guedes, M., Sousa Rodrigues, T., & Ribeiro, J. M. (2013). Correlation between hypertension and functional capacity in older adults: a literature review. Revista de Atencao Primaria a Saude, 16(4), 455-459.
    **Reason: Language exclusion**
22. Barratt, S. L., Davis, R., Sharp, C., & Pauling, J. D. (2020). The prognostic value of cardiopulmonary exercise testing in interstitial lung disease: a systematic review. ERJ Open Research, 6(3).  **Reason: No meta-analysis**
23. Barry Walsh, C., Cahalan, R., Hinman, R. S., & O' Sullivan, K. (2022). Psychometric properties of performance-based measures of physical function administered via telehealth among people with chronic conditions: A systematic review. PloS one, 17(9), e0274349.  **Reason: No meta-analysis**
24. Barry, V. W., Baruth, M., Beets, M.W., Durstine, J. L., Liu, J., & Blair, S. N. (2014) Fitness vs. fatness on allcause mortality: a meta-analysis. Progress in cardiovascular diseases, 56(4), 382-90.  **Reason: Not focused on diagnostic accuracy of CRF (Adult study)**
25. Barry, V. W., Caputo, J. L., & Kang, M. (2018) The Joint Association of Fitness and Fatness on Cardiovascular Disease Mortality: A Meta-Analysis. Progress in cardiovascular diseases, 61(2),136-141.  **Reason: Not focused on diagnostic accuracy of CRF (Adult study)**
26. Bauters, C., & Lemesle, G. (2016). Screening for asymptomatic coronary artery disease in patients with diabetes mellitus: A systematic review and meta-analysis of randomized trials. BMC Cardiovascular Disorders, 16, 90.  **Reason: Not focused on diagnostic accuracy of CRF (Adult study)**
27. Bayonas-Ruiz, A., Munoz-Franco, F. M., Ferrer, V., Perez-Caballero, C., Sabater-Molina, M., Tome-Esteban, M. T., & Bonacasa, B. (2021). Cardiopulmonary Exercise Test in Patients with Hypertrophic Cardiomyopathy: A Systematic Review and Meta-Analysis. Journal of Clinical Medicine, 10(11).  **Reason: Not focused on diagnostic accuracy of CRF (Adult study)**
28. Behrens, G., Niedermaier, T., Berneburg, M., Schmid, D., & Leitzmann, M. F. (2018). Physical activity, cardiorespiratory fitness and risk of cutaneous malignant melanoma: Systematic review and meta-analysis. PLoS ONE, 13(10), e0206087.  **Reason: No meta-analysis**
29. Bell, M., Fotheringham, I., Punekar, Y. S., Riley, J. H., Cockle, S., & Singh, S. J. (2015). Systematic Review of the Association Between Laboratory- and Field-Based Exercise Tests and Lung Function in Patients with Chronic Obstructive Pulmonary Disease. Chronic Obstructive Pulmonary Diseases (Miami, Fla.), 2(4), 321-342.  **Reason: No meta-analysis**
30. Bermejo-Cantarero, A., Alvarez-Bueno, C., Martinez-Vizcaino, V., Garcia-Hermoso, A., Torres-Costoso, A. I., & Sanchez-Lopez, M. (2017). Association between physical activity, sedentary behavior, and fitness with health related quality of life in healthy children and adolescents: A protocol for a systematic review and meta-analysis. Medicine, 96(12), e6407.  **Reason: Not focused on diagnostic accuracy of CRF (Child study)**
31. Bettariga, F., Galvao, D.A., Taaffe, D.R., Bishop, C., Lopez, P., Maestroni, L., Quinto, G., Crainich, U., Verdini, E., Bandini, E. Natalucci, V., & Newton, R. U. (2025). Association of muscle strength and cardiorespiratory fitness with all-cause and cancer-specific mortality in patients diagnosed with cancer: a systematic review with meta-analysis. British Journal of Sports Medicine, 59(10), 722-732.  **Reason: Not focused on diagnostic accuracy of CRF (Adult study)**
32. But-Hadzic, J., Dervisevic, M., Karpljuk, D., Videmsek, M., Dervisevic, E., Paravlic, A., Hadzic, V., & Tomazin, K. (2021). Six-minute walk distance in breast cancer survivors—a systematic review with meta-analysis. International Journal of Environmental Research and Public Health, 18(5), 1-13.  **Reason: Not focused on diagnostic accuracy of CRF (Adult study)**
33. Cadenas-Sanchez, C., Mena-Molina, A., Torres-Lopez, L. V., Migueles, J. H., Rodriguez-Ayllon, M., Lubans, D. R., & Ortega, F. B. (2021). Healthier minds in fitter bodies: a systematic review and meta-analysis of the association between physical fitness and mental health in youth. Sports Medicine, 51(12), 2571-2605.  **Reason: Not focused on diagnostic accuracy of CRF (Child study)**
34. Campos, N. E., Vendrusculo, F. M., DA Costa, G. A., DE Almeida, I. S., Becker, N. A., & Donadio, M. V. F. (2022). The Association of Field Test Outcomes with Peak Oxygen Uptake in Patients with Cystic Fibrosis: A Systematic Review. International journal of exercise science, 15(3), 1381–1394.  **Reason: No meta-analysis**
35. Cantone A, Serenelli M, Sanguettoli F, Maio D, Fabbri G, Dal Passo B, Agostoni P, Grazzi G, Campo G, Rapezzi C. Cardiopulmonary exercise testing predicts prognosis in amyloid cardiomyopathy: a systematic review and meta-analysis. ESC Heart Fail. 2023 Aug;10(4):2740-2744.  **Reason: Not focused on diagnostic accuracy of CRF (Adult study)**
36. Capic, I., Stankovic, M., Spirtovic, O., Corovic, M., Mujanovic, D., Mojsilovic, Z., Jelaska, I., & ZilicFiser, S. (2023). Cardiovascular Fitness in Normal Weight and Obese Children and Adolescents – A Systematic Review of Studies Published After 2000s. International Journal of Morphology, 41(6), 1852–1862.  **Reason: No meta-analysis**
37. Carballada, C., Mihalik, M., Newman-Caro, A., & Walter, A. (2023). Effectiveness of the 6-Minute Walk Test as a Predictive Measure on Hospital Readmission and Mortality in Individuals With Heart Failure: A Systematic Review of the Literature. Journal of Acute Care Physical Therapy, 14(1), 33–44.  **Reason: No meta-analysis**
38. Carrard, J., Guerini, C., Appenzeller-Herzog, C., Infanger, D., Konigstein, K., Streese, L., Hinrichs, T., Hanssen, H., Gallart-Ayala, H., Ivanisevic, J., & Schmidt-Trucksass, A. (2022). The Metabolic Signature of Cardiorespiratory Fitness: A Systematic Review. Sports Medicine (Auckland, N.Z.), 52(3), 527-546.  **Reason: No meta-analysis**
39. Carrasco-Vega, E., Ruiz-Muñoz, M., Cuesta-Vargas, A., Romero-Galisteo, R. P., & González-Sánchez, M. (2022). Individuals with fibromyalgia have a different gait pattern and a reduced walk functional capacity: a systematic review with meta-analysis. PeerJ, 10, e12908.  **Reason: Not focused on diagnostic accuracy of CRF (Adult study)**
40. Cavalcante Neto, J. L., Draghi, T. T. G., Santos, I. W. P. D., Brito, R. D. S., Silva, L. S. D. O., & Lima, U. D. S. (2024). Physical fitness in children with developmental coordination disorder: a systematic review. Physical & Occupational Therapy In Pediatrics, 44(5), 626-655.  **Reason: No meta-analysis**
41. Cavero-Redondo, I., Saz-Lara, A., Bizzozero-Peroni, B., Núñez-Martínez, L., Díaz-Goñi, V., Calero-Paniagua, I., Matínez-García, I. and Pascual-Morena, C. (2024). Accuracy of the 6-minute walk test for assessing functional capacity in patients with heart failure with preserved ejection fraction and other chronic cardiac pathologies: results of the ExIC-FEp trial and a meta-analysis. Sports Medicine-Open, 10(1), 74.  **Reason: Wrong outcomes**
42. Cederberg, K. L. J., Sikes, E. M., Bartolucci, A. A., & Motl, R. W. (2019). Walking endurance in multiple sclerosis: Meta-analysis of six-minute walk test performance. Gait & posture, 73, 147-153.  **Reason: Not focused on diagnostic accuracy of CRF (Adult study)**
43. Cheng, C., Zhang, D., Chen, S., & Duan, G. (2022). The association of cardiorespiratory fitness and the risk of hypertension: a systematic review and dose-response meta-analysis. Journal of Human Hypertension, 36(8), 744– 752.  **Reason: Not focused on diagnostic accuracy of CRF (Adult study)**
44. Cheng, C., Zhang, D., Chen, S., & Duan, G. (2022). The association of cardiorespiratory fitness and the risk of hypertension: a systematic review and dose-response meta-analysis. Journal of human hypertension, 36(8), 744–752.  **Reason: Included in previous search**
45. Cirone, D., Berbrier, D. E., Gibbs, J. C., & Usselman, C. W. (2024). Health-related physical fitness in women with polycystic ovary syndrome versus controls: a systematic review and meta-analysis. Archives of gynecology and obstetrics, 309(1), 17–36.  **Reason: Not focused on diagnostic accuracy of CRF (Adult study)**
46. Cirone, D., Berbrier, D. E., Gibbs, J. C., & Usselman, C. W. (2024). Health-related physical fitness in women with polycystic ovary syndrome versus controls: a systematic review and meta-analysis. Archives of Gynecology and Obstetrics, 309(1), 17-36.  **Reason: Not focused on diagnostic accuracy of CRF (Adult study)**
47. Colcombe, S., & Kramer, A. F. (2003). Fitness Effects on the Cognitive Function of Older Adults: A Meta– Analytic Study. Psychological Science (0956-7976), 14(2), 125-130.  **Reason: No measure of CRF**
48. Copetti Rodriguez, C., de Camargo, E. M., Rodriguez-Añez, C. R., & Siqueira Reis, R. (2020). Physical activity, physical fitness and academic achievement in adolescents: a systematic review. / actividad física, aptitud física y desempeño académico en adolescentes: una revisión sistemática. Revista Brasileira de Medicina do Esporte, 26(5), 441-448.  **Reason: No meta-analysis**
49. Cota, B. C., Suhett, L. G., Leite, N. N., Pereira, P. F., Ribeiro, S. A. V., & Franceschini, S. d. C. C. (2021). Cardiometabolic risk and health behaviours in adolescents with normal-weight obesity: a systematic review. Public Health Nutrition, 24(5), 870-881.  **Reason: No meta-analysis**
50. Cruz-Montecinos, C., Nunez-Cortes, R., Vasconcello-Castillo, L., Solis-Navarro, L., Carrasco-Alonso, B., Calatayud, J., Perez-Alenda, S., & Torres-Castro, R. (2022). Exercise capacity in people with haemophilia: A systematic review. Haemophilia: the official journal of the World Federation of Hemophilia, 28(6), 891-901.  **Reason: No meta-analysis**
51. Cruz-Montecinos, C., Núñez-Cortés, R., Vasconcello-Castillo, L., Solís-Navarro, L., Carrasco-Alonso, B., Calatayud, J., Pérez-Alenda, S., & Torres-Castro, R. (2022). Exercise capacity in people with haemophilia: A systematic review. Haemophilia : the official journal of the World Federation of Hemophilia, 28(6), 891–901.  **Reason: No meta-analysis**
52. D’Ávila, L.B.O., de Lima, A.C.G.B., Milani, M., Milani, J.G.P.O., Cipriano, G.F.B., Le Bihan, D.C., de Castro, I., & Cipriano Jr, G. (2023). Left ventricular global longitudinal strain and cardiorespiratory fitness in patients with heart failure: Systematic review and meta-analysis. Hellenic Journal of Cardiology.  **Reason: Wrong outcomes**
53. da Silva Cunha de Medeiros, R.C., dos Santos, I.K., de Oliveira, A.L.V., de Goes, C.J.D., de Medeiros, J.A., da Silva, T.A.L., de Souza Araujo, J., de Alcântara Varela, P.W., Cobucci, R.N., de Araújo Tinoco Cabral, B.G. & Dantas, P.M.S. (2021). Comparison of muscle strength, aerobic capacity and body composition between healthy adolescents and those living with HIV: A systematic review and meta-analysis. International Journal of Environmental Research and Public Health, 18(11), 5675.  **Reason: Not focused on diagnostic accuracy of CRF (Child study)**
54. de Andrade Goncalves, E. C., Augusto Santos Silva, D., & Gimenes Nunes, H. E. (2015). Prevalence and Factors Associated With Low Aerobic Performance Levels in Adolescents: A Systematic Review. Current Pediatric Reviews, 11(1), 56-70.  **Reason: No meta-analysis**
55. de la Motte, S. J., Gribbin, T. C., Lisman, P., Murphy, K., & Deuster, P. A. (2017). Systematic Review of the Association Between Physical Fitness and Musculoskeletal Injury Risk: Part 2-Muscular Endurance and Muscular Strength. Journal of Strength and Conditioning Research, 31(11), 3218-3234.  **Reason: No meta-analysis**
56. De Visser, H.S., Fast, I., Brunton, N., Arevalo, E., Askin, N., Rabbani, R., Abou-Setta, A.M., & McGavock, J. (2024). Cardiorespiratory fitness and physical activity in pediatric diabetes: a systemic review and meta-analysis. JAMA Network Open, 7(2), e240235-e240235.  **Reason: Not focused on diagnostic accuracy of CRF (Child study)**
57. Demers, K., Bak, M. T. J., Bongers, B. C., de Vries, A. C., Jonkers, D. M. A. E., Pierik, M. J., & Stassen, L. P. S. (2023). Scoping review on health-related physical fitness in patients with inflammatory bowel disease: Assessment, interventions, and future directions. World journal of gastroenterology, 29(38), 5406–5427.  **Reason: No meta-analysis**
58. Dewar, A., Kass, L., Stephens, R. C. M., Tetlow, N., & Desai, T. (2023). Heart Rate Recovery Assessed by Cardiopulmonary Exercise Testing in Patients with Cardiovascular Disease: Relationship with Prognosis. International journal of environmental research and public health, 20(6), 4678.  **Reason: No meta-analysis**
59. Dulfer, K., Helbing, W. A., Duppen, N., & Utens, E. M. W. J. (2014). Associations between exercise capacity, physical activity, and psychosocial functioning in children with congenital heart disease: a systematic review. European Journal of Preventive Cardiology, 21(10), 1200-1215.  **Reason: No meta-analysis**
60. Dun, Y., Wu, S., Cui, N., Thomas, R. J., Olson, T. P., Zhou, N., Li, Q., & Liu, S. (2021). Screening for Asymptomatic Coronary Artery Disease via Exercise Stress Testing in Patients With Type 2 Diabetes Mellitus: A Systematic Review and Meta-Analysis. Frontiers in cardiovascular medicine, 8, 770648.  **Reason:Included in previous search**
61. Durstenfeld, M. S., Sun, K., Tahir, P., Peluso, M. J., Deeks, S. G., Aras, M. A., Grandis, D. J., Long, C. S., Beatty, A., & Hsue, P. Y. (2022). Use of Cardiopulmonary Exercise Testing to Evaluate Long COVID-19 Symptoms in Adults: A Systematic Review and Meta-analysis. JAMA Network Open, 5(10), e2236057.  **Reason: Not focused on diagnostic accuracy of CRF (Adult study)**
62. Eckstein, M. L., Aberer, F., Dobler, F. J. R., Aziz, F., Heise, T., Sourij, H., & Moser, O. (2022). Association of HbA1c with VO2max in Individuals with Type 1 Diabetes: A Systematic Review and Meta-Analysis. Metabolites, 12(11).  **Reason: Not focused on diagnostic accuracy of CRF (Adult study)**
63. Eckstein, M. L., Aberer, F., Dobler, F. J. R., Aziz, F., Heise, T., Sourij, H., & Moser, O. (2022). Association of HbA1c with VO2max in Individuals with Type 1 Diabetes: A Systematic Review and MetaAnalysis. Metabolites, 12(11), 1017.  **Reason: Not focused on diagnostic accuracy of CRF (Adult study)**
64. Etnier, J. L., Nowell, P. M., Landers, D. M., & Sibley, B. A. (2006). A meta-regression to examine the relationship between aerobic fitness and cognitive performance. Brain Research Reviews, 52(1), 119-130.  **Reason: Not focused on diagnostic accuracy of CRF (Adult study)**
65. Ezzatvar, Y., Izquierdo, M., Núñez, J., Calatayud, J., Ramírez-Vélez, R., & García-Hermoso, A. (2021). Cardiorespiratory fitness measured with cardiopulmonary exercise testing and mortality in patients with cardiovascular disease: A systematic review and meta-analysis. Journal of Sport and Health Science, 10(6), 609– 619.  **Reason: Not focused on diagnostic accuracy of CRF (Adult study)**
66. Ezzatvar, Y., Ramírez-Vélez, R., Sáez de Asteasu, M. L., Martínez-Velilla, N., Zambom-Ferraresi, F., Lobelo, F., Izquierdo, M. & García-Hermoso, A. (2021). Cardiorespiratory fitness and all-cause mortality in adults diagnosed with cancer systematic review and meta-analysis. Scandinavian Journal of Medicine & Science in Sports, 31, 1745-1752.  **Reason: Not focused on diagnostic accuracy of CRF (Adult study)**
67. Fan, Y., Gu, X., & Zhang, H. (2019). Prognostic value of six-minute walk distance in patients with heart failure: A meta-analysis. European Journal of Preventive Cardiology, 26(6), 664–667.  **Reason: Not focused on diagnostic accuracy of CRF (Adult study)**
68. Fang, N., Jiang, M., & Fan, Y. (2016). Ideal cardiovascular health metrics and risk of cardiovascular disease or mortality: A meta-analysis. International Journal of Cardiology, 214, 279-283.  **Reason: No measure of CRF**
69. Fernandes Csengeri, L., de Souza, M. K., Soares de Almeida, S., Vanerson Passos Neves, R., de Luca Corrêa, H., Rocha de Moraes, M., Almeida, W. M., de Moraes, M., Gustavo Simões, H., Prestes, J., & dos Santos Rosa, T. (2020). O papel da aptidão aeróbia sobre o paradoxo da obesidade: uma revisão sistemática. / the role of physical fitness in obesity paradox: a systematic review. Revista Brasileira de Ciência e Movimento: RBCM, 28(3), 205-229.  **Reason: Language exclusion**
70. Fernandes M. de Sousa, A., Medeiros, A. R., Del Rosso, S., Stults-Kolehmainen, M., & Boullosa, D. A. (2019). The influence of exercise and physical fitness status on attention: a systematic review. International Review of Sport & Exercise Psychology, 12(1), 202-234.  **Reason: No meta-analysis**
71. Fogelholm, M. (2010). Physical activity, fitness and fatness: relations to mortality, morbidity and disease risk factors. A systematic review. Obesity Reviews: An Official Journal of the International Association for the Study of Obesity, 11(3), 202-221.  **Reason: No meta-analysis**
72. Franklin, J. D., & Graham, M. (2022). Repeated maximal exercise tests of peak oxygen consumption in people with myalgic encephalomyelitis/chronic fatigue syndrome: a systematic review and meta-analysis. Fatigue: Biomedicine, Health and Behavior, 10(3), 119-135.  **Reason: Not focused on diagnostic accuracy of CRF (Adult study)**
73. Franklin, J. D., Atkinson, G., Atkinson, J. M., & Batterham, A. M. (2019). Peak Oxygen Uptake in Chronic Fatigue Syndrome/Myalgic Encephalomyelitis: A Meta-Analysis. International Journal of Sports Medicine, 40(2), 77-87.  **Reason: Not focused on diagnostic accuracy of CRF (Adult study)**
74. Fuentes-Abolafio, I. J., Stubbs, B., Pérez-Belmonte, L. M., Bernal-López, M. R., Gómez-Huelgas, R., & Cuesta-Vargas, A. I. (2020). Physical functional performance and prognosis in patients with heart failure: a systematic review and meta-analysis. BMC Cardiovascular Disorders, 20(1), 512.  **Reason: Not focused on diagnostic accuracy of CRF (Adult study)**
75. García-Hermoso, A., Ramírez-Vélez, R., García-Alonso, Y., Alonso-Martínez, A. M., & Izquierdo, M. (2020). Association of cardiorespiratory fitness levels during youth with health risk later in life: a systematic review and meta-analysis. JAMA pediatrics, 174(10), 952-960.  **Reason: Not focused on diagnostic accuracy of CRF (Child study)**
76. Glue, C., Haveron, R., Smith, M. L., Thiagarajan, P., Edwards, H., Mulligan, H., & Wilkinson, A. (2020). Six-minute walk test values for people with and without long-term conditions in relation to the Walk Score®: a scoping review. Physical Therapy Reviews, 25(5-6), 411-421.  **Reason: No meta-analysis**
77. Gomes-Neto, M., Rodriguez, I., Ledo, A. P., Vieira, J. P. B., & Brites, C. (2018). Muscle Strength and Aerobic Capacity in HIV-Infected Patients: A Systematic Review and Meta-Analysis. Journal of Acquired Immune Deficiency Syndromes (1999), 79(4), 491-500.  **Reason: Not focused on diagnostic accuracy of CRF (Adult study)**
78. Gonçalves, E. C. D. A., Fernandes, R., Alves, C. A. S., Silva, D. A. S., & Trindade, E. B. S. D. M. (2021). Oxygen uptake and indicators of obesity: Meta-analysis including 17,604 adolescents. Revista Brasileira de Medicina do Esporte, 27(6), 621-626.  **Reason: Not focused on diagnostic accuracy of CRF (Child study)**
79. González Valero, G., Zurita Ortega, F., Román Mata, S. S., Pérez Cortés, A. J., Puertas Molero, P., & Chacón Cuberos, R. (2018). Análisis de la capacidad aeróbica como cualidad esencial de la condición física de los estudiantes: Una revisión sistemática. / Analysis of aerobic capacity as an essential quality of students’ physical condition: A systematic review. Retos: Nuevas Perspectivas de Educación Física, Deporte y Recreación, 34, 395- 402.  **Reason: No meta-analysis**
80. Gottarde, L. A. F., Vitor, G. B. B., Martini, F. A. N., & de Oliveira, R. G. (2021). Atividade física, aptidão física e dor lombar em adultos jovens: revisão sistemática de evidências observacionais. Fisioterapia Brasil, 22(6), 931-950.  **Reason:** Language exclusion
81. Grao-Cruces, A., Ruiz-Ariza, A., De La Torre-Cruz, M. J., & Martínez-López, E. J. (2018). Students with excess weight obtain lower physical fitness test scores in physical education than normal weight pairs: Myth or reality? Journal of Physical Education and Sport, 18(3), 1562-1571.  **Reason: No meta-analysis**
82. Grässler, B., Hökelmann, A., & Cabral, R. H. (2020). Resting Heart Rate Variability As A Possible Marker of Cognitive Decline. Kinesiology, 52(1), 72-84.  **Reason: No meta-analysis**
83. Guo, A. A., Zeng, K., Bushati, Y., Kim, P., Zhong, W., Chalasani, V., & Winter, M. (2024). Cardiopulmonary exercise testing prior to radical cystectomy: a systematic review and meta‐analysis. BJU international, 134, 22-29.  **Reason: Wrong patient population**
84. Guo, L., & Zhang, S. (2017). Association between ideal cardiovascular health metrics and risk of cardiovascular events or mortality: A meta-analysis of prospective studies. Clinical cardiology, 40(12), 1339-1346.  **Reason: No measure of CRF**
85. Han, M., Qie, R., Shi, X., Yang, Y., Lu, J., Hu, F., Zhang, M., Zhang, Z., Hu, D., & Zhao, Y. (2022). Cardiorespiratory fitness and mortality from all causes, cardiovascular disease and cancer: dose-response metaanalysis of cohort studies. British Journal of Sports Medicine, 56(13), 733–739.  **Reason: Not focused on diagnostic accuracy of CRF (Adult study)**
86. Hansen, D., Marinus, N., Remans, M., Courtois, I., Cools, F., Calsius, J., Massa, G., & Takken, T. (2014). Exercise tolerance in obese vs. lean adolescents: a systematic review and meta‐analysis. obesity reviews, 15(11), 894-904.  **Reason: Not focused on diagnostic accuracy of CRF (Child study)**
87. Heggelund, J., Vancampfort, D., Tacchi, M. J., Morken, G., & Scott, J. (2020). Is there an association between cardiorespiratory fitness and stage of illness in psychotic disorders? A systematic review and meta-analysis. Acta Psychiatrica Scandinavica, 141(3), 190-205.  **Reason: Not focused on diagnostic accuracy of CRF (Adult study)**
88. Hou, Y., Yan, L., Mao, J., Wang, X., Li, L., Zhong, C., Liu, H., & Zhao, Z. (2018). The application value of 6-minute walking test in clinical evaluation of chronic heart failure. Chinese Journal of Evidence-Based Medicine, 18(9), 907-917.  **Reason:** Language exclusion
89. Huerta-Uribe, N., Hormazábal-Aguayo, I. A., Izquierdo, M., & García-Hermoso, A. (2023). Youth with type 1 diabetes mellitus are more inactive and sedentary than apparently healthy peers: A systematic review and meta-analysis. Diabetes research and clinical practice, 200, 110697.   **Reason: Not focused on diagnostic accuracy of CRF (Child study)**
90. Huerta-Uribe, N., Ramirez-Velez, R., Izquierdo, M., & Garcia-Hermoso, A. (2023). Association between physical activity, sedentary behavior and physical fitness and glycated hemoglobin in youth with type 1 diabetes: a systematic review and meta-analysis. Sports medicine, 53(1), 111-123.  **Reason: Not focused on diagnostic accuracy of CRF (Child study)**
91. Huerta-Uribe, N., Ramírez-Vélez, R., Izquierdo, M., & García-Hermoso, A. (2023). Association Between Physical Activity, Sedentary Behavior and Physical Fitness and Glycated Hemoglobin in Youth with Type 1 Diabetes: A Systematic Review and Meta-analysis. Sports medicine (Auckland, N.Z.), 53(1), 111–123.  **Reason: Included in previous search**
92. Husoy, A., Dalene, K. E., Steene-Johannessen, J., Anderssen, S. A., Ekelund, U., & Tarp, J. (2021). Effect modification by cardiorespiratory fitness on the association between physical activity and cardiometabolic health in youth: A systematic review. Journal of Sports Sciences, 39(8), 845-853.  **Reason: No meta-analysis**
93. Jiménez-Pavón, D., Lavie, C. J., & Blair, S. N. (2019). The role of cardiorespiratory fitness on the risk of sudden cardiac death at the population level: A systematic review and meta-analysis of the available evidence. Progress in Cardiovascular Diseases, 62(3), 279–287.  **Reason: Not focused on diagnostic accuracy of CRF (Adult study)**
94. Kandola, A., Ashdown-Franks, G., Stubbs, B., Osborn, D. P. J., & Hayes, J. F. (2019). The association between cardiorespiratory fitness and the incidence of common mental health disorders: A systematic review and meta-analysis. Journal of Affective Disorders, 257, 748–757.  **Reason: Not focused on diagnostic accuracy of CRF (Adult study)**
95. Ketels, M., Cillekens, B., Clays, E., Huysmans, M. A., De Bacquer, D., Holtermann, A., Troiano, R.P., Mork, P.J., Krokstad, S., Völzke, H., Dörr, M., Bahls, M., Ittermann, T., Clausen, J., Jensen, M.T., Kauhanen, J., Voutilainen, A., Wanner, M., Bopp, M., van Mechelen, W., van der Beek, A.J., & Coenen, P. (2025). Exploring the ‘fit for work’principle: The association between occupational physical activity, cardio-respiratory fitness, and mortality–a meta-analysis of male worker data. Scandinavian Journal of Work, Environment & Health, 51(3), 159.  **Reason: Not focused on diagnostic accuracy of CRF (Adult study)**
96. Kodama, S., Saito, K., Tanaka, S., Maki, M., Yachi, Y., Asumi, M., Sugawara, A., Totsuka, K., Shimano, H., Ohashi, Y., Yamada, N., & Sone, H. (2009). Cardiorespiratory fitness as a quantitative predictor of all-cause mortality and cardiovascular events in healthy men and women: a meta-analysis. JAMA, 301(19), 2024–2035.  **Reason: Not focused on diagnostic accuracy of CRF (Adult study)**
97. Kunutsor SK, Isiozor NM, Myers J, Seidu S, Khunti K, Laukkanen JA. Baseline and usual cardiorespiratory fitness and the risk of chronic kidney disease: A prospective study and meta-analysis of published observational cohort studies. Geroscience. 2023 Jun;45(3):1761-1774.  **Reason: Not focused on diagnostic accuracy of CRF (Adult study)**
98. Kunutsor, S. K., Kaminsky, L. A., Lehoczki, A., & Laukkanen, J. A. (2024). Unraveling the link between cardiorespiratory fitness and cancer: a state-of-the-art review. GeroScience, 46(6), 5559–5585.  **Reason: No meta-analysis**
99. Kunutsor, S. K., Kurl, S., & Laukkanen, J. A. (2024). Cardiorespiratory fitness, atrial fibrillation and stroke: a review of the evidence in 2024. Expert review of cardiovascular therapy, 22(9), 493–508.  **Reason: No meta-analysis**
100. Kyröläinen, H., Santtila, M., Nindl, B. C., & Vasankari, T. (2010). Physical fitness profiles of young men associations between physical fitness, obesity and health. Sports Medicine, 40(11), 907-920.  **Reason: No meta-analysis**
101. Lachman, S., Terbraak, M. S., Limpens, J., Jorstad, H., Lucas, C., Scholte Op Reimer, W., Boekholdt, S. M., Ter Riet, G., Peters, R. J. G. (2018). The prognostic value of heart rate recovery in patients with coronary artery disease: A systematic review and meta-analysis. American Heart Journal, 199, 163-169.  **Reason: Not focused on diagnostic accuracy of CRF (Adult study)**
102. Langeskov-Christensen, M., Heine, M., Kwakkel, G., & Dalgas, U. (2015). Aerobic capacity in persons with multiple sclerosis: a systematic review and meta-analysis. Sports Medicine (Auckland, N.Z.), 45(6), 905-923.  **Reason: Not focused on diagnostic accuracy of CRF (Adult study)**
103. Lardon, A., Leboeuf-Yde, C., & Le Scanff, C. (2015). Is back pain during childhood or adolescence associated with muscle strength, muscle endurance or aerobic capacity: three systematic literature reviews with one meta-analysis. Chiropractic & Manual Therapies, 23, 21.  **Reason: No meta-analysis**
104. Laukkanen, J. A., Isiozor, N. M., & Kunutsor, S. K. (2022). Objectively Assessed Cardiorespiratory Fitness and All-Cause Mortality Risk: An Updated Meta-analysis of 37 Cohort Studies Involving 2,258,029 Participants. Mayo Clinic Proceedings, 97(6), 1054–1073.  **Reason: Not focused on diagnostic accuracy of CRF (Adult study)**
105. Lee, C. H. A., Kong, J. C., Ismail, H., Riedel, B., & Heriot, A. (2018). Systematic Review and Meta-analysis of Objective Assessment of Physical Fitness in Patients Undergoing Colorectal Cancer Surgery. Diseases of the Colon and Rectum, 61(3), 400-409.  **Reason: Not focused on diagnostic accuracy of CRF (Adult study)**
106. Lee, J. (2020). Influences of Cardiovascular Fitness and Body Fatness on the Risk of Metabolic Syndrome: A Systematic Review and Meta-Analysis. American journal of health promotion: AJHP, 34(7), 796-805.  **Reason: Not focused on diagnostic accuracy of CRF (Adult study)**
107. Lee, J. Cardiorespiratory Fitness, Physical Activity, Walking Speed, Lack of Participation in Leisure Activities, and Lung Cancer Mortality: A Systematic Review and Meta-Analysis of Prospective Cohort Studies. (2021). Cancer Nursing, 44(6), 453-464.  **Reason: Not focused on diagnostic accuracy of CRF (Adult study)**
108. Lee J. (2021). Cardiorespiratory Fitness, Physical Activity, Walking Speed, Lack of Participation in Leisure Activities, and Lung Cancer Mortality: A Systematic Review and Meta-Analysis of Prospective Cohort Studies. Cancer nursing, 44(6), 453–464.  **Reason: Included in previous search**
109. Lee, J. Influence of Cardiorespiratory Fitness on Risk of Dementia and Dementia Mortality: A Systematic Review and Meta-Analysis of Prospective Cohort Studies. (2021). Journal of Aging and Physical Activity, 29(5), 878–885.  **Reason: Not focused on diagnostic accuracy of CRF (Adult study)**
110. Liu, C., He, Y., Venn, A. J., Jose, M. D., & Tian, J. (2023). Childhood modifiable risk factors and later life chronic kidney disease: a systematic review. BMC nephrology, 24(1), 184.  **Reason: No meta-analysis**
111. Lona, G., Hauser, C., Köchli, S., Infanger, D., Endes, K., Schmidt-Trucksäss, A., & Hanssen, H. (2022). Association of blood pressure, obesity and physical activity with arterial stiffness in children: a systematic review and meta-analysis. Pediatric Research, 91(3), 502-512.  **Reason: Not focused on diagnostic accuracy of CRF (Child study)**
112. Macedo, A. C. P., Schaan, C. W., Bock, P. M., Pinto, M. B., Botton, C. E., Umpierre, D., & Schaan, B. D. (2023). Cardiorespiratory fitness in individuals with type 2 diabetes mellitus: a systematic review and metaanalysis. Archives of endocrinology and metabolism, 67(5), e230040.  **Reason: Not focused on diagnostic accuracy of CRF (Adult study)**
113. Madigan, S., Proudman, S., Schembri, D., Davies, H., & Adams, R. (2024). Use of exercise tests in screening for pulmonary arterial hypertension in systemic sclerosis: A systematic literature review. Journal of scleroderma and related disorders, 9(1), 50–58.  **Reason: No meta-analysis**
114. Maleki, S., Hendrikse, J., Chye, Y., Caeyenberghs, K., Coxon, J. P., Oldham, S., Suo, C., & Yucel, M. (2022). Associations of cardiorespiratory fitness and exercise with brain white matter in healthy adults: A systematic review and meta-analysis. Brain imaging and behavior, 16(5), 2402-2425.  **Reason: Not focused on diagnostic accuracy of CRF (Adult study)**
115. Marques, A., Gouveira, E. R., Peralta, M., Martins, J., Venturini, J., Henriques-Neto, D., & Sarmento, H. (2020). Cardiorespiratory fitness and telomere length: a systematic review. Journal of Sports Sciences, 38(14), 1690-1697.  **Reason: No meta-analysis**
116. Marques, A., Santos, D. A., Hillman, C. H., & Sardinha, L. B. (2018). How does academic achievement relate to cardiorespiratory fitness, self-reported physical activity and objectively reported physical activity: a systematic review in children and adolescents aged 6-18 years. British Journal of Sports Medicine, 52(16), 1039.  **Reason: No meta-analysis**
117. Martins, R., Wamosy, R. M. G., Cardoso, J., Schivinski, C. I. S., & Beltrame, T. S. (2021). Evaluation of cardiopulmonary system outcomes in children with developmental coordination disorder: A systematic review. Human Movement Science, 80, 102888.  **Reason: No meta-analysis**
118. McAlister, K. L., Zhang, D., Moore, K. N., Chapman, T. M., Zink, J., & Belcher, B. R. (2022). A Systematic Review of the Associations of Adiposity and Cardiorespiratory Fitness With Arterial Structure and Function in Nonclinical Children and Adolescents. Pediatric Exercise Science, 1-12.  **Reason: No meta-analysis**
119. McAlister, K. L., Zhang, D., Moore, K. N., Chapman, T. M., Zink, J., & Belcher, B. R. (2022). A Systematic Review of the Associations of Adiposity and Cardiorespiratory Fitness With Arterial Structure and Function in Nonclinical Children and Adolescents. Pediatric exercise science, 35(3), 174–185.  **Reason: No meta-analysis**
120. Medrano-Ureña, M. R., Ortega-Ruiz, R., & Benítez-Sillero, J. D. (2020). Physical fitness, exercise selfefficacy, and quality of life in adulthood: A systematic review. International Journal of Environmental Research and Public Health, 17(17), 1-19.  **Reason: No meta-analysis**
121. Mehanna, E., Hamik, A., & Josephson, R. A. (2016). Cardiorespiratory Fitness and Atherosclerosis: Recent Data and Future Directions. Current Atherosclerosis Reports, 18(5).  **Reason: No meta-analysis**
122. Mendelson, M., Marillier, M., Bailly, S., Flore, P., Borel, J. C., Vivodtzev, I., Doutreleau, S., Tamisier, R., Pépin, J. L., & Verges, S. (2018). Maximal exercise capacity in patients with obstructive sleep apnoea syndrome: A systematic review and meta-analysis. European Respiratory Journal, 51(6).  **Reason: Not focused on diagnostic accuracy of CRF (Adult study)**
123. Millard-Stafford, M., Becasen, J. S., Beets, M. W., Nihiser, A. J., Lee, S. M., & Fulton, J. E. (2013). Is Physical Fitness Associated with Health in Overweight and Obese Youth? A Systematic Review. Kinesiology Review (Champaign, Ill.), 2(4), 233-247.  **Reason: No meta-analysis**
124. Mintjens, S., Menting, M. D., Daams, J. G., van Poppel, M. N. M., Roseboom, T. J., & Gemke, R. J. B. J. (2018). Cardiorespiratory Fitness in Childhood and Adolescence Affects Future Cardiovascular Risk Factors: A Systematic Review of Longitudinal Studies. Sports Medicine (Auckland, N.Z.), 48(11), 2577-2605.  **Reason: No meta-analysis**
125. Montero, D. (2015). The association of cardiorespiratory fitness with endothelial or smooth muscle vasodilator function. European Journal of Preventive Cardiology, 22(9), 1200-1211.  **Reason: No meta-analysis**
126. Moore, D., Jung, M., Hillman, C. H., Kang, M., & Loprinzi, P. D. (2022). Interrelationships between exercise, functional connectivity, and cognition among healthy adults: A systematic review. Psychophysiology, 59(6), e14014.  **Reason: No meta-analysis**
127. Morishita, S., Tsubaki, A., & Shirai, N. (2017). Physical function was related to mortality in patients with chronic kidney disease and dialysis. Hemodialysis International, 21(4), 483-489.  **Reason: No meta-analysis**
128. Morris, D. R., Rodriguez, A. J., Moxon, J. V., Cunningham, M. A., McDermott, M. M., Myers, J., Leeper, N. J., Jones, R. E., & Golledge, J. (2014). Association of lower extremity performance with cardiovascular and all-cause mortality in patients with peripheral artery disease: a systematic review and meta-analysis. Journal of the American Heart Association, 3(4), e001105.  **Reason: Not focused on diagnostic accuracy of CRF (Adult study)**
129. Morrison, J. D., & Mayer, L. (2017). Physical activity and cognitive function in adults with multiple sclerosis: an integrative review. Disability & Rehabilitation, 39(19), 1909-1920.  **Reason: No meta-analysis**
130. Morton-Holtham, L., Swann, N., Brewer, J., & Moir, H. J. (2021). A systematic review of physical activity and physical fitness in sarcoidosis. Translational Sports Medicine, 4(6), 741-755.  **Reason: No meta-analysis**
131. Mucke, M., Ludyga, S., Colledge, F., & Gerber, M. (2018). Influence of Regular Physical Activity and Fitness on Stress Reactivity as Measured with the Trier Social Stress Test Protocol: A Systematic Review. Sports medicine (Auckland, N.Z.), 48(11), 2607-2622.  **Reason: No meta-analysis**
132. Munsterman, T., Takken, T., & Wittink, H. (2012). Are persons with rheumatoid arthritis deconditioned? A review of physical activity and aerobic capacity. BMC Musculoskeletal Disorders, 13, 202.  **Reason: No meta-analysis**
133. Nativi-Nicolau, J., Yilmaz, A., Dasgupta, N., Macey, R., Cochrane, J., Peatman, J., Summers, C., Luth, J., & Zolty, R. (2024). Six-minute walk test as clinical end point in cardiomyopathy clinical trials, including ATTR-CM: a systematic literature review. Journal of comparative effectiveness research, 13(7), e230158.  **Reason: No meta-analysis**
134. Navarrete-Villanueva, D., Gomez-Cabello, A., Marin-Puyalto, J., Moreno, L. A., Vicente-Rodriguez, G., & Casajus, J. A. (2021). Frailty and Physical Fitness in Elderly People: A Systematic Review and Meta-analysis. Sports medicine (Auckland, N.Z.), 51(1), 143-160.  **Reason: Not focused on diagnostic accuracy of CRF (Adult study)**
135. Neil-Sztramko, S. E., Kirkham, A. A., Hung, S. H., Niksirat, N., Nishikawa, K., & Campbell, K. L. (2014). Aerobic capacity and upper limb strength are reduced in women diagnosed with breast cancer: a systematic review. Journal of Physiotherapy, 60(4), 189-200.  **Reason: No meta-analysis**
136. Núñez-Cortés, R., Padilla-Acevedo, P., Vergara-Peña, F., Mollà-Casanova, S., Espinoza-Bravo, C., TorresCastro, R., & Cruz-Montecinos, C. (2022). Clinical assessment of balance and functional impairments in people with stable chronic obstructive pulmonary disease: a systematic review and meta-analysis. ERJ open research, 8(4), 00164-2022.  **Reason: Not focused on diagnostic accuracy of CRF (Adult study)**
137. O’Brien, M. W., Petterson, J. L., Wu, Y., Bray, N. W., & Kimmerly, D. S. (2022). What is the impact of aerobic fitness and movement interventions on low-flow-mediated vasoconstriction? A systematic review of observational and intervention studies. Vascular Medicine (United Kingdom), 27(2), 193-202.  **Reason: No meta-analysis**
138. Oh, S., You, J., & Kim, Y. W. (2022). Physical Fitness for Depression in Adolescents and Adults: A MetaAnalysis. Iranian Journal of Public Health, 51(11), 2425-2434.  **Reason: Not focused on diagnostic accuracy of CRF (Adult study)**
139. Oh, S., You, J., & Kim, Y. W. (2022). Physical Fitness for Depression in Adolescents and Adults: A Meta Analysis. Iranian journal of public health, 51(11), 2425–2434.  **Reason: Not focused on diagnostic accuracy of CRF (Adult study)**
140. Oliveira, A., Jacome, C., & Marques, A. (2014). Physical fitness and exercise training on individuals with spina bifida: a systematic review. Research in Developmental Disabilities, 35(5), 1119-1136.  **Reason: No meta-analysis**
141. Oliveira, R. G. D., & Guedes, D. P. (2016). Physical activity, sedentary behavior, cardiorespiratory fitness and metabolic syndrome in adolescents: systematic review and meta-analysis of observational evidence. PloS one, 11(12), e0168503.  **Reason: Not focused on diagnostic accuracy of CRF (Child study)**
142. Ortega, F. B., Cadenas-Sanchez, C., Migueles, J. H., Labayen, I., Ruiz, J. R., Sui, X., Blair, S. N., Martínez-Vizcaino, V., Lavie, C. J., & Martínez-Vizcaino, V. (2018). Role of Physical Activity and Fitness in the Characterization and Prognosis of the Metabolically Healthy Obesity Phenotype: A Systematic Review and Metaanalysis. Progress in Cardiovascular Diseases, 61(2), 190-205.  **Reason: Not focused on diagnostic accuracy of CRF (Adult study)**
143. Papasavvas, T., Alhashemi, M., & Micklewright, D. (2017). Association Between Depressive Symptoms and Exercise Capacity in Patients With Heart Disease: A META-ANALYSIS. Journal of Cardiopulmonary Rehabilitation and Prevention, 37(4), 239-249.  **Reason: Not focused on diagnostic accuracy of CRF (Adult study)**
144. Papasavvas, T., Bonow, R., Alhashemi, M., & Micklewright, D. (2016). Depression Symptom Severity and Cardiorespiratory Fitness in Healthy and Depressed Adults: A Systematic Review and Meta-Analysis. Sports Medicine, 46(2), 219-230.  **Reason: Not focused on diagnostic accuracy of CRF (Adult study)**
145. Parry, S. M., Nalamalapu, S. R., Nunna, K., Rabiee, A., Friedman, L. A., Colantuoni, E., Needham, D. M., & Dinglas, V. D. (2021). Six-Minute Walk Distance After Critical Illness: A Systematic Review and MetaAnalysis. Journal of Intensive Care Medicine, 36(3), 343-351.  **Reason: Not focused on diagnostic accuracy of CRF (Adult study)**
146. Pavasini, R., Guralnik, J., Brown, J. C., di Bari, M., Cesari, M., Landi, F., Vaes, B., Legrand, D., Verghese, J., Wang, C., Stenholm, S., Ferrucci, L., Lai, J. C., Bartes, A. A., Espaulella, J., Ferrer, M., Lim, J.-Y., Ensrud, K. E., Cawthon, P., Turusheva, A., Frolova, E., Rolland, Y., Lauwers, V., Corsonello, A., Kirk, G. D., Ferrari, R., Volpato, S., & Campo, G. (2016). Short Physical Performance Battery and all-cause mortality: systematic review and meta-analysis. BMC Medicine, 14(1), 215.  **Reason: No measure of CRF**
147. Peel, A. B., Thomas, S. M., Dittus, K., Jones, L. W., & Lakoski, S. G. (2014). Cardiorespiratory fitness in breast cancer patients: A call for normative values. Journal of the American Heart Association, 3(1), e000432.  **Reason:Not a systematic review**
148. Pella, E., Sgouropoulou, V., Theodorakopoulou, M., Iatridi, F., Boutou, A., Karpetas, A., Papagianni, A., Sarafidis, P., & Dimitroulas, T. (2023). Cardiorespiratory fitness assessed with cardiopulmonary exercise testing in patients with juvenile idiopathic arthritis: a systematic review and meta-analysis. Rheumatology, 62(11), 3526-3533.  **Reason: Not focused on diagnostic accuracy of CRF (Child study)**
149. Pella, E., Theodorakopoulou, M. P., Boutou, A. K., Alexandrou, M. E., Bakaloudi, D. R., Sarridou, D., Boulmpou, A., Papadopoulos, C., Papagianni, A., & Sarafidis, P. (2022). Cardiopulmonary reserve examined with cardiopulmonary exercise testing in individuals with chronic kidney disease: A systematic review and metaanalysis. Annals of Physical and Rehabilitation Medicine, 65(5).  **Reason: Not focused on diagnostic accuracy of CRF (Adult study)**
150. Peytremann-Bridevaux, I., Staeger, P., Bridevaux, P. O., Ghali, W. A., & Burnand, B. (2008). Effectiveness of Chronic Obstructive Pulmonary Disease-Management Programs: Systematic Review and Meta-Analysis. American Journal of Medicine, 121(5), 433-443.e434.  **Reason: Not focused on diagnostic accuracy of CRF (Adult study)**
151. Pozuelo-Carrascosa, D. P., Alvarez-Bueno, C., Cavero-Redondo, I., Morais, S., Lee, I. M., & MartínezVizcaíno, V. (2019). Cardiorespiratory fitness and site-specific risk of cancer in men: A systematic review and meta-analysis. European Journal of Cancer (Oxford, England : 1990), 113, 58–68.  **Reason: Not focused on diagnostic accuracy of CRF (Adult study)**
152. Prado, J. C. D., Guedes, D. P., Dias, P. H. G., Stabelini Neto, A., & Oliveira, R. G. (2024). Associations Between Cardiorespiratory Fitness and Metabolic Syndrome in Adolescents: A Systematic Review and Meta-Analysis. Metabolites, 14(11), 635.  **Reason: Not focused on diagnostic accuracy of CRF (Child study)**
153. Pressler, A. (2017). A run a day keeps lipids at bay? Regular exercise as a treatment of dyslipidaemias. Deutsche Zeitschrift fur Sportmedizin, 68(11), 253-260.  **Reason: No meta-analysis**
154. Punekar, Y. S., Riley, J. H., Lloyd, E., Driessen, M., & Singh, S. J. (2017). Systematic review of the association between exercise tests and patient-reported outcomes in patients with chronic obstructive pulmonary disease. International Journal of Chronic Obstructive Pulmonary Disease, 12, 2487-2506.  **Reason: No meta-analysis**
155. Qiu, S., Cai, X., Liu, J., Yang, B., Sun, Z., Zügel, M., Steinacker, J. M., & Schumann, U. (2019). Association Between Cardiorespiratory Fitness and Risk of Heart Failure: A Meta-Analysis. Journal of Cardiac Failure, 25(7), 537–544.  **Reason: Not focused on diagnostic accuracy of CRF (Adult study)**
156. Qiu, S., Cai, X., Sun, Z., Wu, T., & Schumann, U. (2021). Is estimated cardiorespiratory fitness an effective predictor for cardiovascular and all-cause mortality? A meta-analysis. Atherosclerosis, 330, 22–28.  **Reason: Not focused on diagnostic accuracy of CRF (Adult study)**
157. Qiu, S., Cai, X., Yang, B., Du, Z., Cai, M., Sun, Z., Zügel, M., Michael Steinacker, J., & Schumann, U. (2019). Association Between Cardiorespiratory Fitness and Risk of Type 2 Diabetes: A Meta-Analysis. Obesity (Silver Spring, Md.), 27(2), 315–324.  **Reason: Not focused on diagnostic accuracy of CRF (Adult study)**
158. Ramirez-Velez, R., Saavedra, J. M., Lobelo, F., Celis-Morales, C. A., Pozo-Cruz, B. D., & GarciaHermoso, A. (2018). Ideal Cardiovascular Health and Incident Cardiovascular Disease Among Adults: A Systematic Review and Meta-analysis. Mayo Clinic Proceedings, 93(11), 1589-1599.  **Reason: No measure of CRF**
159. Rauner, A., Mess, F., & Woll, A. (2013). The relationship between physical activity, physical fitness and overweight in adolescents: A systematic review of studies published in or after 2000. BMC Pediatrics, 13(1).  **Reason: No meta-analysis**
160. Rigdon, B., & Loprinzi, P. D. (2019). The Association of Cardiorespiratory Fitness on Memory Function: Systematic Review. Medicina (Kaunas, Lithuania), 55(5).  **Reason: No meta-analysis**
161. Rivilis, I., Hay, J., Cairney, J., Klentrou, P., Liu, J., & Faught, B. E. (2011). Physical activity and fitness in children with developmental coordination disorder: A systematic review. Research in Developmental Disabilities, 32(3), 894-910.  **Reason: No meta-analysis**
162. Rocha, V., Paixão, C., & Marques, A. (2022). Physical activity, exercise capacity and mortality risk in people with interstitial lung disease: A systematic review and meta-analysis. Journal of Science And Medicine in Sport, 25(11), 903–910.  **Reason: Not focused on diagnostic accuracy of CRF (Adult study)**
163. Rocha, V., Paixão, C., & Marques, A. (2022). Physical activity, exercise capacity and mortality risk in people with interstitial lung disease: A systematic review and meta-analysis. Journal of science and medicine in sport, 25(11), 903–910.  **Reason: Included in previous search**
164. Rodrigues, T., Raposo, S. C., Brito, D., & Lopes, L. R. (2021). Prognostic relevance of exercise testing in hypertrophic cardiomyopathy. A systematic review. International Journal of Cardiology, 339, 83-92.  **Reason: No meta-analysis**
165. Rodriguez, C. C., de Camargo, E. M., Rodriguez-Añez, C. R., & Reis, R. S. (2020). Physical activity, physical fitness and academic achievement in adolescents: A systematic review. Revista Brasileira de Medicina do Esporte, 26(5), 441-448.  **Reason: No meta-analysis**
166. Ruiz, J. R., Cavero-Redondo, I., Ortega, F. B., Welk, G. J., Andersen, L. B., & Martinez-Vizcaino, V. (2016). Cardiorespiratory fitness cut points to avoid cardiovascular disease risk in children and adolescents; what level of fitness should raise a red flag? A systematic review and meta-analysis. British Journal of Sports Medicine, 50(23), 1451-1458.  **Reason: Not focused on diagnostic accuracy of CRF (Adult study)**
167. Ruiz-Ariza, A., Grao-Cruces, A., de Loureiro, N. E. M., & Martínez-López, E. J. (2017). Influence of physical fitness on cognitive and academic performance in adolescents: A systematic review from 2005–2015. International Review of Sport & Exercise Psychology, 10(1), 108-133.  **Reason: No meta-analysis**
168. Santana, C. C. A., Azevedo, L. B., Cattuzzo, M. T., Hill, J. O., Andrade, L. P., & Prado, W. L. (2017). Physical fitness and academic performance in youth: A systematic review. Scandinavian journal of medicine & science in sports, 27(6), 579-603.  **Reason: No meta-analysis**
169. Savarese, G., Paolillo, S., Costanzo, P., D'Amore, C., Cecere, M., Losco, T., Musella, F., Gargiulo, P., Marciano, C., & Perrone-Filardi, P. (2012). Do changes of 6-minute walk distance predict clinical events in patients with pulmonary arterial hypertension?: A meta-analysis of 22 randomized trials. Journal of the American College of Cardiology, 60(13), 1192-1201.  **Reason: Not focused on diagnostic accuracy of CRF (Adult study)**
170. Sawada, S., Miyachi, M., Murakami, H., Kawakami, R., Tanaka, S., Ishikawa-Takata, K., Tabata, I., Oida, Y., Oguma, Y., Miyatake, N., Okamoto, T., Tsukamoto, K., Tanaka, H., Lee, I., & Blair, S. (2012). Dose-response relationship between cardiorespiratory fitness and morbidity/mortality: a systematic review and meta-analysis. Journal of Science & Medicine in Sport, 15, S25-S25.  **Reason: Conference abstract**
171. Schaan, C. W., de Macedo, A. C. P., Sbruzzi, G., Umpierre, D., Schaan, B. D., & Pellanda, L. C. (2017). Functional capacity in congenital heart disease: A systematic review and Meta-Analysis. Arquivos Brasileiros de Cardiologia, 109(4), 357-367.  **Reason: Not focused on diagnostic accuracy of CRF (Child study)**
172. Schmid, D., & Leitzmann, M. F. (2015). Cardiorespiratory fitness as predictor of cancer mortality: a systematic review and meta-analysis. Annals of oncology : official journal of the European Society for Medical Oncology, 26(2), 272-278.  **Reason: Not a systematic review**
173. Schmid, V., Paterson, S., Weinkauf, C., Wang, J., Tomczak, C., Niederseer, D., Vontobel, J., Nelson, M.D., Forman, D.E., Halle, M., Foulkes, S., & Haykowsky, M. Peak Oxygen Uptake in Older Adults with Heart Failure a Systematic Review and Meta-Analysis. Available at SSRN 5085547.  **Reason: Not focused on diagnostic accuracy of CRF (Adult study)**
174. Schmidt-Andersen, P., Stage, A., Pouplier, A., Bastholm, L. H., Müller, K. G., Larsen, A., Ness, K. K., Larsen, H. B., Christensen, J., & Fridh, M. K. (2024). Physical capacity in children and adolescents with newly diagnosed cancer: A systematic review and meta-analysis. Pediatric blood & cancer, 71(1), e30746.  **Reason: Not focused on diagnostic accuracy of CRF (Child study)**
175. Schmidt‐Andersen, P., Stage, A., Pouplier, A., Bastholm, L.H., Müller, K.G., Larsen, A., Ness, K.K., Larsen, H.B., Christensen, J., & Fridh, M.K. (2024). Physical capacity in children and adolescents with newly diagnosed cancer: A systematic review and meta‐analysis. Pediatric Blood & Cancer, 71(1), e30746.  **Reason: Not focused on diagnostic accuracy of CRF (Child study)**
176. Schuch, F. B., Vancampfort, D., Sui, X., Rosenbaum, S., Firth, J., Richards, J., Ward, P. B., & Stubbs, B. (2016). Are lower levels of cardiorespiratory fitness associated with incident depression? A systematic review of prospective cohort studies. Preventive Medicine, 93, 159–165.  **Reason: Not focused on diagnostic accuracy of CRF (Adult study)**
177. Silveira, J. F. C., López-Gil, J. F., Reuter, C. P., Sehn, A. P., Borfe, L., Carvas Junior, N., Pfeiffer, K. A., Guerra, P. H., Andersen, L. B., Garcia-Hermoso, A., & Reis Gaya, A. (2025). Mediation of obesity-related variables in the association between physical fitness and cardiometabolic risk in children and adolescents: a systematic review and meta-analysis. BMJ open sport & exercise medicine, 11(2), e002366.  **Reason: Not focused on diagnostic accuracy of CRF (Child study)**
178. Singh, B., Cadenas-Sanchez, C., da Costa, B. G. G., Castro-Piñero, J., Chaput, J. P., Cuenca-García, M., Maher, C., Marín-Jiménez, N., McGrath, R., Molina-García, P., Myers, J., Gower, B., Ortega, F. B., Lang, J. J., & Tomkinson, G. R. (2024). Comparison of objectively measured and estimated cardiorespiratory fitness to predict all-cause and cardiovascular disease mortality in adults: A systematic review and meta-analysis of 42 studies representing 35 cohorts and 3.8 million observations. Journal of sport and health science, 14, 100986. Advance online publication.  **Reason: Not focused on diagnostic accuracy of CRF (Adult study)**
179. Sivakumar, J., Sivakumar, H., Read, M., Sinclair, R. C. F., Snowden, C. P., & Hii, M. W. (2020). The Role of Cardiopulmonary Exercise Testing as a Risk Assessment Tool in Patients Undergoing Oesophagectomy: A Systematic Review and Meta-analysis. Annals of Surgical Oncology, 27(10), 3783-3796.  **Reason: Not focused on diagnostic accuracy of CRF (Adult study)**
180. Sonaglioni, A., Nicolosi, G. L., Bruno, A., & Lombardo, M. (2025). Accuracy of noninvasive screening exercise tests for detecting coronary artery disease in symptomatic patients with mitral valve prolapse: a systematic review. Journal of cardiovascular medicine (Hagerstown, Md.), 26(3), 122–130.  **Reason: No meta-analysis**
181. Soydara, C., Jurgens, C.Y., & Lewis, G.D. (2023). Postexercise Oxygen Uptake Recovery Delay Among Patients with Heart Failure: A systematic review. Heart and Mind, 7(1), 40–44.  **Reason: No meta-analysis**
182. Steiman De Visser, H., Fast, I., Brunton, N., Arevalo, E., Askin, N., Rabbani, R., Abou-Setta, A. M., & McGavock, J. (2024). Cardiorespiratory Fitness and Physical Activity in Pediatric Diabetes: A Systemic Review and Meta-Analysis. JAMA network open, 7(2), e240235.  **Reason: Wrong outcomes**
183. Tacchi, M. J., Heggelund, J., & Scott, J. (2019). Predictive validity of objective measures of physical fitness for the new onset of mental disorders in adolescents and young adults. Early Intervention in Psychiatry, 13(6), 1310-1318.  **Reason: Not focused on diagnostic accuracy of CRF (Adult study)**
184. Tarp, J., Støle, A. P., Blond, K., & Grøntved, A. (2019). Cardiorespiratory fitness, muscular strength and risk of type 2 diabetes: a systematic review and meta-analysis. Diabetologia, 62(7), 1129–1142.  **Reason: Not focused on diagnostic accuracy of CRF (Adult study)**
185. Te Hoonte, F., Spronk, M., Sun, Q., Wu, K., Fan, S., Wang, Z., Bots, M. L., Van der Schouw, Y. T., Uijl, A., & Vernooij, R. W. M. (2023). Ideal cardiovascular health and cardiovascular related events: a systematic review and meta-analysis. European journal of preventive cardiology, zwad405. Advance online publication.  **Reason: No measure of CRF**
186. Thrue, C., Hvid, L. G., Gamborg, M., Dawes, H., Dalgas, U., & Langeskov-Christensen, M. (2022). Aerobic capacity in persons with Parkinson's disease: a systematic review. Disability and Rehabilitation, 1-13.  **Reason: No meta-analysis**
187. Thrue, C., Hvid, L. G., Gamborg, M., Dawes, H., Dalgas, U., & Langeskov-Christensen, M. (2023). Aerobic capacity in persons with Parkinson's disease: a systematic review. Disability and rehabilitation, 45(15), 2409–2421.  **Reason: No meta-analysis**
188. Udholm, S., Aldweib, N., Hjortdal, V. E., & Veldtman, G. R. (2018). Prognostic power of cardiopulmonary exercise testing in Fontan patients: a systematic review. Open heart, 5(1), e000812.  **Reason: No meta-analysis**
189. Van Waelvelde, H., Vanden Wyngaert, K., Mariën, T., Baeyens, D., & Calders, P. (2020). The relation between children's aerobic fitness and executive functions: A systematic review. Infant and Child Development, 29(3).  **Reason: No meta-analysis**
190. Vancampfort, D., Mugisha, J., Rosenbaum, S., Firth, J., De Hert, M., Probst, M., & Stubbs, B. (2016). Cardiorespiratory fitness levels and moderators in people with HIV: A systematic review and meta-analysis. Preventive Medicine, 93, 106-114.  **Reason: Not focused on diagnostic accuracy of CRF (Adult study)**
191. Vancampfort, D., Rosenbaum, S., Probst, M., Soundy, A., Mitchell, A. J., De Hert, M., & Stubbs, B. (2015). Promotion of cardiorespiratory fitness in schizophrenia: a clinical overview and meta-analysis. Acta Psychiatrica Scandinavica, 132(2), 131-143.  **Reason: Not focused on diagnostic accuracy of CRF (Adult study)**
192. Vancampfort, D., Rosenbaum, S., Schuch, F., Ward, P., Richards, J., Mugisha, J., Probst, M., & Stubbs, B. (2017). Cardiorespiratory Fitness in Severe Mental Illness: A Systematic Review and Meta-analysis. Sports Medicine, 47(2), 343-352.  **Reason: Not focused on diagnostic accuracy of CRF (Adult study)**
193. Vancampfort, D., Stubbs, B., Richards, J., Ward, P. B., Firth, J., Schuch, F. B., & Rosenbaum, S. (2017). Physical fitness in people with posttraumatic stress disorder: a systematic review. Disability and Rehabilitation, 39(24), 2461-2467.  **Reason: No meta-analysis**
194. Vancampfort, D., Ward, P. B., & Stubbs, B. (2019). Physical fitness levels and moderators in people with epilepsy: A systematic review and meta-analysis. Epilepsy & Behavior : E&B, 99, 106448.  **Reason: Not focused on diagnostic accuracy of CRF (Adult study)**
195. Vandecasteele, E., De Pauw, M., De Keyser, F., Decuman, S., Deschepper, E., Piette, Y., Brusselle, G., & Smith, V. (2016). Six-minute walk test in systemic sclerosis: A systematic review and meta-analysis. International Journal of Cardiology, 212, 265-273.  **Reason: Not focused on diagnostic accuracy of CRF (Adult study)**
196. Vendrusculo, F. M., Heinzmann-Filho, J. P., da Silva, J. S., Ruiz, M. P., & Donadio, M. V. F. (2019). Peak oxygen uptake and mortality in cystic fibrosis: systematic review and meta-analysis. Respiratory care, 64(1), 91-98.  **Reason: Not focused on diagnostic accuracy of CRF (Child study)**
197. Verweij, N. M., Schiphorst, A. H. W., Pronk, A., van den Bos, F., & Hamaker, M. E. (2016). Physical performance measures for predicting outcome in cancer patients: a systematic review. Acta Oncologica, 55(12), 1386-1391.  **Reason: No meta-analysis**
198. Vetsch, T., Eggmann, S., Jardot, F., von Gernler, M., Engel, D., Beilstein, C. M., Wuethrich, P. Y., Eser, P., & Wilhelm, M. (2024). Ventilatory efficiency as a prognostic factor for postoperative complications in patients undergoing elective major surgery: a systematic review. British journal of anaesthesia, 133(1), 178–189.  **Reason: No meta-analysis**
199. Villaseca-Rojas, Y., Varela-Melo, J., Torres-Castro, R., Vasconcello-Castillo, L., Mazzucco, G., Vilaró, J., & Blanco, I. (2022). Exercise capacity in children and adolescents with congenital heart disease: a systematic review and meta-analysis. Frontiers in Cardiovascular Medicine, 9, 874700.  **Reason: Not focused on diagnostic accuracy of CRF (Child study)**
200. Villaseca-Rojas, Y., Varela-Melo, J., Torres-Castro, R., Vasconcello-Castillo, L., Mazzucco, G., Vilaró, J., & Blanco, I. (2022). Exercise Capacity in Children and Adolescents with Congenital Heart Disease: A Systematic Review and Meta-Analysis. Frontiers in cardiovascular medicine, 9, 874700.  **Reason:Included in previous search**
201. Wadey, C. A., Weston, M. E., Dorobantu, D. M., Pieles, G. E., Stuart, G., Barker, A. R., Taylor, R. S., & Williams, C. A. (2022). The role of cardiopulmonary exercise testing in predicting mortality and morbidity in people with congenital heart disease: a systematic review and meta-analysis. European Journal of Preventive Cardiology, 29(3), 513-533.  **Reason: Not focused on diagnostic accuracy of CRF (Adult study)**
202. Wang, Y., Li, F., Cheng, Y., Gu, L., & Xie, Z. (2020). Cardiorespiratory fitness as a quantitative predictor of the risk of stroke: a dose-response meta-analysis. Journal of Neurology, 267(2), 491–501.  **Reason: Not focused on diagnostic accuracy of CRF (Adult study)**
203. Weeldreyer, N. R., De Guzman, J. C., Paterson, C., Allen, J. D., Gaesser, G. A., & Angadi, S. S. (2025). Cardiorespiratory fitness, body mass index and mortality: a systematic review and meta-analysis. British journal of sports medicine, 59(5), 339–346.  **Reason: Not focused on diagnostic accuracy of CRF (Adult study)**
204. Weinkauf, K., Fyfe, E., Hewitt, D., Wang, J., Kennedy, M., Pituskin, E., La Gerche, A., Foulkes, S. J., & Haykowsky, M. J. (2024). Cardiorespiratory Fitness in Childhood Cancer Survivors: A Systematic Review and Meta-Analysis. European journal of preventive cardiology, zwae317. Advance online publication.  **Reason: Not focused on diagnostic accuracy of CRF (Child study)**
205. Wijnhoud, E. J., Bos, A. M. M. E., Buizer, A. I., & Beckerman, H. (2024). Aerobic fitness in children with cerebral palsy compared to typically developing peers: A systematic review and meta-analysis. Brazilian journal of physical therapy, 28(6), 101142.  **Reason: Not focused on diagnostic accuracy of CRF (Child study)**
206. Wong, T. E., Majumdar, S., Adams, E., Bergman, S., Damiano, M. L., Deutsche, J., Recht, M., & Healthy Weight Working Group. (2011). Overweight and obesity in hemophilia: a systematic review of the literature. American Journal of Preventive Medicine, 41(6 Suppl 4), S369-375.  **Reason: No meta-analysis**
207. Xue, Z., Zhou, Y., Wu, C., Lin, J., Liu, X., Yu, P., & Zhu, W. (2020). Dose-response relationship of cardiorespiratory fitness with incident atrial fibrillation. Heart Failure Reviews, 25(3), 419–425.  **Reason: Not focused on diagnostic accuracy of CRF (Adult study)**
208. Yamamoto, S., Yamaga, T., Nishie, K., Sakai, Y., Ishida, T., Oka, K., Ikegami, S., & Horiuchi, H. (2020). Impact of physical performance on prognosis among patients with heart failure: Systematic review and metaanalysis. Journal of Cardiology, 76(2), 139–146.  **Reason: Not focused on diagnostic accuracy of CRF (Adult study)**
209. Yang, L., He, Y., & Li, X. (2023). Physical function and all-cause mortality in patients with chronic kidney disease and end-stage renal disease: a systematic review and meta-analysis. International Urology and Nephrology, 55(5), 1219–1228.  **Reason: Not focused on diagnostic accuracy of CRF (Adult study)**
210. Yang, L., He, Y., & Li, X. (2023). Physical function and all-cause mortality in patients with chronic kidney disease and end-stage renal disease: a systematic review and meta-analysis. International urology and nephrology, 55(5), 1219–1228.  **Reason: Included in previous search**
211. Yerrakalva, D., Mullis, R., & Mant, J. (2015). The associations of "fatness," "fitness," and physical activity with all-cause mortality in older adults: A systematic review. Obesity (Silver Spring, Md.), 23(10), 1944-1956.   **Reason: No meta-analysis**
212. Yildiz Kabak, V., Calders, P., Duger, T., Mohammed, J., & van Breda, E. (2019). Short and long-term impairments of cardiopulmonary fitness level in previous childhood cancer cases: a systematic review. Supportive care in cancer: official journal of the Multinational Association of Supportive Care in Cancer, 27(1), 69-86.   **Reason: No meta-analysis**
213. Yin, X., Huang, J., & Zhao, Y. (2024). Association between elevated preoperative VE/VCO2 slope and increased mortality following major surgery: a meta-analysis. Minerva anestesiologica, 90(9), 805–813.  **Reason: Not focused on diagnostic accuracy of CRF (Child study)**
214. Zaccardi, F., O'Donovan, G., Webb, D. R., Yates, T., Kurl, S., Khunti, K., Davies, M. J., & Laukkanen, J. A. (2015). Cardiorespiratory fitness and risk of type 2 diabetes mellitus: A 23-year cohort study and a metaanalysis of prospective studies. Atherosclerosis, 243(1), 131–137.  **Reason: Not focused on diagnostic accuracy of CRF (Adult study)**
215. Zambolin, F., Duro-Ocana, P., Faisal, A., Bagley, L., Gregory, W. J., Jones, A. W., & McPhee, J. S. (2022). Fibromyalgia and Chronic Fatigue Syndromes: A systematic review and meta-analysis of cardiorespiratory fitness and neuromuscular function compared with healthy individuals. PLoS ONE, 17(10), e0276009.   **Reason: Not focused on diagnostic accuracy of CRF (Adult study)**
216. Zambolin, F., Duro-Ocana, P., Faisal, A., Bagley, L., Gregory, W. J., Jones, A. W., & McPhee, J. S. (2022). Fibromyalgia and Chronic Fatigue Syndromes: A systematic review and meta-analysis of cardiorespiratory fitness and neuromuscular function compared with healthy individuals. PloS one, 17(10), e0276009.  **Reason: Not focused on diagnostic accuracy of CRF (Adult study)**
217. Zhang, F., Bai, Y., Huang, L., & Zhong, Y. (2025). Cardiopulmonary fitness in children/adolescents with chronic kidney disease and the impact of exercise training: a systematic review and meta-analysis of observational study and randomized controlled trials. Annals of medicine, 57(1), 2458197.  **Reason: Not focused on diagnostic accuracy of CRF (Child study)**
218. Zielinska, D., Bellwon, J., Rynkiewicz, A., & Elkady, M. A. (2013). Prognostic value of the six-minute walk test in heart failure patients undergoing cardiac surgery: a literature review. Rehabilitation research and practice, 2013, 965494.  **Reason: No meta-analysis**

**eAppendix 4.** GRADE certainty assessment at the outcome level.

| Study | Measure/index | Risk of Bias | Imprecision | Inconsistency | Indirectness | Publication Bias | Large magnitude of effect | GRADE at CRF measure level | GRADE at health outcome level |
| --- | --- | --- | --- | --- | --- | --- | --- | --- | --- |
|  | **Severe cardiovascular events** | | | | | | | | |
| Cahalin et al. [21] | Ventilatory efficiency (VE/VCO_2_ slope) | Serious | Not serious | Not serious | Serious | Not serious | Large effect | High | ⨁⨁⨁⭘  Moderate |
| Cahalin et al. [21] | VO_2_ peak | Serious | Not serious | Very serious | Serious | Not serious | Large effect | Low |  |
| Cahalin et al. [21] | Ventilatory function (exercise oscillatory ventilation) | Serious | Not serious | Not serious | Serious | Not serious | Large effect | High |  |
| Cahalin et al. [21] | Ventilatory efficiency (VO_2_ efficiency slope) | Serious | Not serious | Not serious | Serious | Not serious | Large effect | High |  |
|  | **Cardiovascular disease risk** | | | | | | | | |
| Ruiz et al. [23] | Maximal oxygen consumption (VO_2max_) | Serious | Not serious | Very serious | Serious | Not serious | Large effect | Very Low-to-Low | ⨁⨁⭘⭘  Low |
|  | **Coronary artery disease** | | | | | | | | |
| Sun et al. [24] | Exercise performance (treadmill exercise test) | Serious | Not serious | Not serious | Very serious | Not serious | Large effect | Low-to-Moderate | ⨁⭘⭘⭘  Very Low |
| Dun et al. [22] | Exercise performance (exercise stress test) | Very serious | Not serious | Very serious | Serious | Not serious | Small effect | Very low-to-Low |  |
| Banerjee et al. [25] | Exercise stress test (all combined) | Very serious | Very serious | Very serious | Serious | Very serious | NR | Very Low |  |
| Banerjee et al. [25] | Treadmill ECG | Very serious | Very serious | Very serious | Serious | Very serious | NR | Very Low |  |
| Banerjee et al. [25] | Treadmill echocardiogram | Serious | Very serious | Very serious | Serious | Not serious | NR | Very Low |  |
| Banerjee et al. [25] | Bicycle ECG | Very serious | Very serious | Very serious | Serious | Very serious | NR | Very Low |  |
| Banerjee et al. [25] | Bicycle Echocardiogram | Serious | Very serious | Very serious | Serious | Not serious | NR | Very Low |  |
| Banerjee et al. [25] | Myocardial perfusion imaging | Very serious | Very serious | Very serious | Serious | Very serious | NR | Very Low |  |
|  | **Mitochondrial disorders** | | | | | | | | |
| El Guessabi et al. [26] | Absolute load lactate stress test (LSTA) | Not serious | Not serious | Not serious | Serious | Not serious | Large effect | High | ⨁⨁⨁⨁  High |
| El Guessabi et al. [26[ | Relative load lactate stress test (LSTR) | Not serious | Not serious | Not serious | Serious | Not serious | Large effect | High |  |
| El Guessabi et al. [26] | Overall LST (defined as LSTA and LSTR studies combined) | Not serious | Not serious | Not serious | Serious | Not serious | Large effect | High |  |

*Abbreviations:* NR not reported, ECG electrocardiogram

Summary of Decision Rules: GRADE Assessments for Certainty of Evidence

| **Domain** | **Judgment** | **Scoring** | **Criteria** |
| --- | --- | --- | --- |
| **Risk of Bias* (1)** | No serious RoB | 0 | - Most (>80%) primary studies assessed as having a low risk of bias (e.g., score of low on QUADAS or QUADAS 2). |
|  | Serious RoB | $-$0.5 point | - All primary studies were assessed to be at low to moderate risk of bias (e.g., score of low on QUADAS or QUADAS 2) with a potential for bias arising from a lack of clarity on eligibility criteria, measurement of exposure or outcome, confounding, and/or loss to follow-up. |
|  | Very serious RoB | $-$1 point | - At least one of the primary studies was assessed to be of high risk of bias (e.g., score of high or unclear on QUADAS or QUADAS 2), but the relative contribution of each study to the overall results will be considered for assessing the overall level of bias. - Risk of biases were not assessed and there was evidence of not having appropriate eligibility criteria and the populations are not generalizable, there is a serious flaw in the measurement of the exposure (i.e., CRF measurement method) and/or outcome (i.e., health outcomes), there is a failure to adequately control for confounding (i.e., age, sex). Biases related to generalizability, measurement of exposure, inadequate follow-up time (i.e., <2 years), and adequate control for confounding are considered the most important for the study. |
| **Inconsistency (2)** | No serious inconsistency | 0 | - Point estimates are similar across studies with overlap of confidence intervals. - Statistical tests for heterogeneity are not significant and the I^2^ is low to moderate (i.e., <50%). |
|  | Serious inconsistency | $-$0.5 point | - Heterogeneity is high (i.e., ≥50%) but can be explained by important sub-group differences (e.g., test types, ways of measuring the primary outcomes). |
|  | Very serious inconsistency | $-$1 point | - Point estimates vary widely across studies. - Confidence intervals show minimal or no overlap. - The statistical test for heterogeneity is significant at p<0.05 and cannot be explained by sub-group analyses. - The I^2^ is large (i.e., ≥50%) but ***cannot*** be explained by important sub-group differences (e.g., test types, ways of measuring the primary outcomes). |
| **Indirectness (3)** | No serious indirectness | 0 | - There was good global representation within the primary studies (e.g., variety of populations, sex distribution). - The tests used to assess health outcome/s all used objective measurement (e.g., cardiopulmonary exercise testing). - There was relatively equal distribution of males and females captured in the studies and a broad/representativeness range of ethnicities captured in the analysed cohorts. |
|  | Serious indirectness | $-$0.5 point | The studies were limited by having one of the following:   - Limited global representation of primary studies with consideration for their relative contribution to the estimate. - Considerable inclusion of participants <18 years or only one sex captured in the studies or only one ethnicity captured in the studies. |
|  | Very serious indirectness | $-$1 point | The studies were limited by having two or more of the following:   - Limited global representation of primary studies. - Considerable inclusion of participants <18 years or only one sex captured in the studies. - Considerable inclusion of self-reported tests used to assess CRF across the studies. |
| **Imprecision (4)** | No serious imprecision | 0 | - The 95% CIs for the pooled dOR estimates do not include 1.0. |
|  | Serious imprecision | $-$0.5 point | - The 95% CIs for the pooled estimates include 1.0 but the CIs include important benefit or harm (i.e., 10%). |
|  | Very serious imprecision | $-$1 point | - There are very few events and the CIs around the estimates of effect include both appreciable benefit and appreciable harm (e.g., dOR = 0.96, 95% CI: 0.56, 1.69). |
| **Publication Bias (5)** | No serious publication bias | 0 | - There is no suspected evidence of publication bias as reported in the systematic review based on Egger’s test and/or visual inspection of funnel plots. - There were an insufficient number of included studies to assess publication bias (<10 studies). - The search strategy appears comprehensive. |
|  | Serious publication bias | $-$0.5 point | The studies were limited by having at least one of the following:   - There is evidence of publication bias arising from asymmetrical rather than symmetrical funnel plots, or if statistical tests of asymmetry are positive. - Publication bias was not assessed and there were ≥10 studies. - The search strategy is not comprehensive. |
|  | Very serious publication bias | $-$1 point | The studies were limited by having two or more of the following:   - There is evidence of publication bias arising from asymmetrical rather than symmetrical funnel plots, or if statistical tests of asymmetry are positive. - Publication bias was not assessed and there were ≥10 studies. - The search strategy is not comprehensive. |

CI – confidence interval, CRF – cardiorespiratory fitness, QUADAS – quality assessment of diagnostic accuracy studies, RoB – risk of bias, dOR – diagnostic odds ratio.

*Risk of bias will be based on the quality assessment of the primary studies included in the systematic reviews. If a RoB assessment is not provided within the systematic review, a de novo RoB assessment will be conducted using the QUADAS 2 tool.

- The certainty of the evidence was upgraded, if there was no cause to downgrade, and there was evidence of a large magnitude of effect from meta-analyses with sufficiently narrow confidence intervals that do not overlap the chosen thresholds* (+1 large effect), evidence of a dose-response gradient (+1), or when all residual confounding would decrease the magnitude of the effect (+1). *Large effect is defined as dOR values of 2.0, 3.0, and 4.012 low, moderate, and high.
- All outcomes started at a rating of ‘high’ certainty per PERSIST guidelines (Ardern, C.L., Büttner, F., Andrade, R., Weir, A., Ashe, M.C., Holden, S., Impellizzeri, F.M., Delahunt, E., Dijkstra, H.P., Mathieson, S. and Rathleff, M.S., 2022. Implementing the 27 PRISMA 2020 Statement items for systematic reviews in the sport and exercise medicine, musculoskeletal rehabilitation and sports science fields: the PERSiST (implementing Prisma in Exercise, Rehabilitation, Sport medicine and SporTs science) guidance. British journal of sports medicine, 56(4), pp.175-195.)
- The quality of the evidence **per outcome** can be interpreted as follows (6):

**High:** we are confident that the true magnitude of effect between CRF and the cardiovascular outcome lies close to the effect estimated in the meta-analysis and further research is unlikely to change our confidence in the magnitude of effect.

**Moderate:** we are moderately confident that the true magnitude of effect between CRF and the cardiovascular outcome is likely to be close to the effect estimated in the meta-analysis, but there is a possibility that it is substantially different; further research is likely to have an important impact on the confidence in the direction of association and may change the direction of association.

**Low:** we have limited confidence; the true magnitude of effect between CRF and the cardiovascular outcome may be substantially different from the estimate.

**Very low:** we have very little confidence; the true magnitude of effect between CRF and the cardiovascular outcome may be substantially different from the estimate.
